# Stable maintenance of MERVL-positive embryonic stem cells reveals sustained transcriptional programs and enhancer remodeling

**DOI:** 10.1016/j.jbc.2026.113166

**Published:** 2026-05-15

**Authors:** Rui Geng, Benjamin L. Kidder

**Affiliations:** 1 Department of Oncology, Wayne State University School of Medicine, Detroit, Michigan, USA; 2 Karmanos Cancer Institute, Wayne State University School of Medicine, Detroit, Michigan, USA

**Keywords:** 2CLC, chromatin, ChIP-seq, embryonic stem cell, epigenetics, MERVL, LTR, pluripotency, super enhancer, two-cell, totipotency, RNA-seq

## Abstract

Mouse embryonic stem cells (ESCs) occasionally transit into a rare two-cell-like (2C) state characterized by transient activation of endogenous retroviruses such as MERVL and expression of 2C-specific genes including the Zscan4 cluster. These 2C-like cells resemble early blastomeres and display expanded developmental potential, but their unstable and sporadic nature has hindered mechanistic studies. Here, we demonstrate the transiently stable maintenance of MERVL-positive ESCs that exhibit persistent MERVL expression and activation of 2C-associated genes. Live-cell imaging revealed uniform and sustained MERVL activity in these MERVL-positive ESCs, contrasting with the heterogeneous and transient expression observed in conventional ESCs. Transcriptome profiling demonstrated robust induction of 2C-specific regulatory networks, and embryoid body differentiation combined with machine learning uncovered increased lineage variability and altered developmental trajectories. Single-cell RNA sequencing revealed clear separation of control ESCs from MERVL-positive populations and redistribution across distinct transcriptional states, with Red and Mosaic lines showing graded shifts within a shared transcriptional manifold. Epigenomic profiling further revealed distinct chromatin states, specialized super-enhancer landscapes, and active enhancer marking at MERVL loci. Together, these findings demonstrate that stable maintenance of MERVL-positive ESCs is achievable *in vitro*, providing a powerful model to dissect endogenous retroviral element -driven transcriptional regulation, epigenomic remodeling, and 2C-like transcriptional and epigenetic programs.

Mouse embryonic stem cells (ESCs), derived from the inner cell mass of blastocyst-stage embryos, are pluripotent and capable of generating derivatives of all three embryonic germ layers. In contrast, totipotent cells such as zygotes and two-cell (2C) stage blastomeres can give rise to both embryonic and extraembryonic lineages (1). Understanding how cells transition into the totipotent state is a central question in developmental biology, as it defines the earliest developmental potential. In mice, zygotic genome activation (ZGA) peaks at the 2C stage (2), coinciding with robust expression of repetitive sequences such as major satellites (3, 4), endogenous retroviral elements (ERVs) including MERVL (4, 5, 6), and numerous 2C-specific genes (7, 8, 9).

ESC cultures occasionally generate rare two-cell-like cells that transiently resemble blastomeres and co-express MERVL with genes such as Zscan4 (6, 10, 11). These cells revert rapidly, and MERVL expression is silenced beyond the 2C stage (12, 13). Many 2C-specific genes are directly driven by MERVL long terminal repeat (LTR) promoters, highlighting a functional role for retroelements in totipotent-like gene activation (4, 11). Transposable elements account for more than half of the mammalian genome, with ERV-derived LTR retrotransposons representing a major class (14). While ESCs are characterized by transcriptional and epigenetic heterogeneity (15, 16, 17), evidence suggests that epigenetic mechanisms such as histone modifications contribute to MERVL activation. Within ESC cultures, MERVL-positive cells represent a transient and unstable subpopulation, and MERVL-positive ESCs display elevated histone acetylation (4, 18), and perturbations that alter chromatin states can increase or decrease the frequency of MERVL-expressing cells (4, 18, 19, 20, 21, 22, 23, 24, 25, 26). These findings link chromatin remodeling to ERV activity, yet mechanistic analysis has been hindered by the fleeting nature of the 2C-like state.

A barrier in the field has been the inability to stably capture a homogeneous population of 2CLCs without extensive perturbation of the epigenome. As a result, the transcriptional and chromatin mechanisms sustaining totipotent-like identity have remained incompletely defined. We hypothesized that stable maintenance of MERVL-positive ESCs would overcome this limitation by enabling long-term analysis of MERVL-driven transcription and chromatin states. Here, we report the stable maintenance of MERVL-positive ESCs (sMERVL ESCs), characterized by persistent expression of MERVL and 2C-specific genes. Transcriptome and epigenome profiling revealed a distinct regulatory network, altered differentiation potential, and unique enhancer landscapes marked by activating histone modifications and super enhancer (SE) formation. The sMERVL ESC model provides a tractable system for dissecting ERV-driven transcriptional regulation, epigenomic remodeling, and totipotent-like developmental potential.

## Results

### Identification and prospective isolation of transiently stable MERVL-positive ESCs

**Figure 1:**
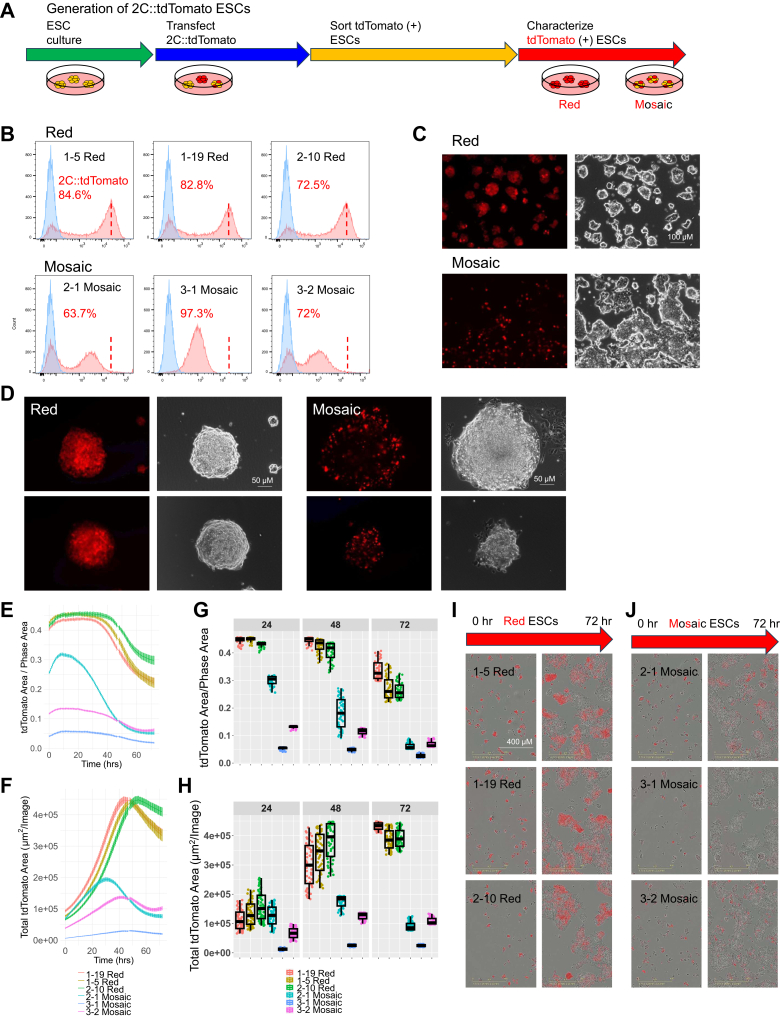
**Identification and characterization of stable MERVL-positive ESCs.***A*, schematic of the experimental workflow. ESCs were transfected with a modified 2C::tdTomato reporter vector (Addgene #40281) and subjected to FACS sorting to isolate MERVL-positive cells. The resulting populations carried stable MERVL promoter-driven tdTomato integrations, enabling visualization and prospective isolation of 2C-like cells. *B*, the 2C::tdTomato reporter was used to detect MERVL expression, enabling fluorescence-activated cell sorting of sMERVL ESCs. *Red* (*top*) and Mosaic (*bottom*) ESC colonies contained a higher proportion of MERVL-positive cells compared with conventional ESCs (see Fig. S1). Live reporter-positive cells were defined as tdTomato+/DAPI− following exclusion of DAPI-positive (nonviable) cells. *C*, post-sort analysis of ESCs showed variable MERVL-positivity in *red* and *mosaic* populations. Fluorescent (2C::tdTomato; *left*) and bright field (*right*) images are shown. *D*, representative fluorescent and bright field images of *red* (*left*) and *mosaic* (*right*) MERVL-positive ESCs. *E* and *H*, sequential imaging of *red* and *mosaic* clones under self-renewal conditions, with quantification of tdTomato area relative to phase area or total tdTomato area (μ^2^/image). These analyses show that *red* sMERVL ESCs maintain a higher and more stable fraction of MERVL-positive cells compared with Mosaic sMERVL ESCs during the initial 48 h. After 48 h, 60 to 70% of cells were reporter-positive in fluorescent images. *I* and *J*, representative bright field and fluorescent images at 0 and 72 h, demonstrating sustained MERVL expression in *red* cells compared with the more sporadic expression observed in *mosaic* colonies. ESC, embryonic stem cell; sMERVL ESC, stable maintenance of MERVL-positive ESC.

To visualize and track sMERVL ESCs, we generated stable integrations of a MERVL promoter-driven 2C::tdTomato reporter (4) (Fig. 1*A*; See Experimental procedures). Flow cytometry of bulk cultures revealed ∼15–16% reporter-positive cells (Fig. S1), representing the presence of a MERVL-positive subpopulation under self-renewal conditions. We then used fluorescence-activated cell sorting (FACS) to prospectively isolate 2C::tdTomato + cells and clonally expand reporter-positive colonies. Following the initial FACS isolation, reporter-positive cells were plated at clonal density and expanded without iterative re-sorting. Elevated tdTomato-positive fractions (typically ∼60–70% in Red-derived clones) were observed after the first replating passage, indicating stable propagation of reporter activity from the initial sort.

Two reproducible phenotypic classes emerged among reporter-positive colonies: (i) “Mosaic” colonies with heterogeneous, generally lower reporter signal, and (ii) “Red” colonies with uniformly higher reporter intensity. Flow cytometry of expanded clones confirmed that both classes maintained substantial reporter-positive fractions, with Red clones exhibiting markedly higher MERVL reporter levels (Fig. 1, *B* and *C*). In some red clones, up to 84% of cells were reporter-positive, far exceeding conventional ESCs, whereas mosaic clones contained fewer reporter-positive cells and at lower intensity (Fig. 1, *B* and *C*). Higher-magnification micrographs illustrate the contrasting expression patterns (Fig. 1*D*).

Live-cell imaging over 72 h showed that MERVL activation in mosaic colonies was dynamic, with transitions into and out of the positive state, whereas Red colonies displayed sustained, homogeneous reporter expression. Quantification by total tdTomato area per image and tdTomato/phase area ratio demonstrated that Red sMERVL ESCs retained higher and more stable reporter levels throughout the time course (Fig. 1, *E–H*). After the first replating passage following FACS isolation, ∼60 to 70% of cells in Red-derived clones were reporter-positive. During subsequent live-cell imaging (∼40–48 h), this elevated fraction was maintained, and by 72 h most cells in Red clones expressed the reporter, whereas Mosaic clones remained lower on average (Fig. 1, *E–H*). Representative fields at 0 h and 72 h are shown for both classes (Fig. 1, *I* and *J*). Notably, at the 72-h time point, partial reduction and increased heterogeneity of tdTomato signal are observed in some Red colonies (Fig. 1*I*); however, this interval extends beyond standard ESC passaging conditions, where increased culture density can impact self-renewal and reporter activity, and therefore is interpreted with caution. Based on their persistence and uniformity over defined imaging intervals (24–72 h) and across multiple passages, we define these Red and Mosaic reporter-positive lines collectively as transiently stable MERVL-positive ESCs.

### Clonal stability of single-cell–derived red and mosaic sMERVL ESCs

To determine whether the Red and Mosaic phenotypes represent stable lineage states or interconvertible populations, we performed single-cell recloning experiments from independently derived Red and Mosaic MERVL-positive ESC lines. Individual tdTomato-positive cells from representative Red and Mosaic clones were isolated by FACS and plated at clonal density. Emerging colonies were expanded and reanalyzed by flow cytometry and live-cell imaging.

Single-cell–derived colonies from Red lines predominantly regenerated colonies with uniformly high tdTomato intensity, retaining the characteristic Red phenotype. FACS analysis demonstrated that these secondary Red-derived clones maintained elevated fractions of reporter-positive cells relative to Mosaic-derived clones.(Fig. S2*A*). In contrast, single-cell–derived colonies from Mosaic lines largely reestablished heterogeneous, lower-intensity reporter expression profiles consistent with the original mosaic phenotype (Fig. S2*A*).

Longitudinal live-cell imaging further confirmed these differences. Red-derived reclones exhibited sustained and homogeneous tdTomato signal over time, whereas mosaic-derived reclones displayed dynamic fluctuations in reporter activity, with transitions between tdTomato-positive and -negative states (Fig. S2, *B* and *C*). Quantification of total tdTomato area and tdTomato-to-phase ratios demonstrated preservation of the original expression patterns across passages.

Although some variability in the percentage of reporter-positive cells was observed following recloning, the overall phenotypic identity of Red *versus* Mosaic lines was retained. We did not observe systematic or directional interconversion of Red-derived clones into Mosaic-like populations or *vice versa* under standard self-renewal conditions within the time frames examined, although transitions over longer durations or under different conditions cannot be excluded. These results indicate that Red and Mosaic MERVL-positive ESCs represent reproducibly propagating clonal states rather than transient fluctuations within a single unstable population. Importantly, these phenotypes were maintained across multiple passages following single-cell derivation, supporting the reproducible propagation of these states under standard ESC culture conditions.

### Reproducible derivation and stable maintenance of MERVL-positive ESCs in an independent ESC background

To test the reproducibility and generality of stable MERVL-positive cell maintenance, we repeated the reporter-based isolation strategy in an independent ESC line. R1 ESCs were transfected with the MERVL promoter driven 2C::tdTomato reporter, followed by FACS to isolate tdTomato-positive cells. Following transfection with the MERVL promoter-driven 2C::tdTomato reporter and exclusion of DAPI-positive (nonviable) cells, live reporter-positive populations were defined as tdTomato+/DAPI−, revealing a distinct tdTomato-positive subpopulation (∼8%) (Fig. S3). Single cells were clonally expanded to establish independent reporter-positive ESC lines.

Flow cytometric analysis revealed two reproducible classes of tdTomato-positive R1 ESC clones. These consisted of Red clones with uniformly high tdTomato fluorescence and Mosaic clones exhibiting heterogeneous reporter expression across the population (Fig. S4, *A* and *B*). Quantification across multiple independently derived clones demonstrated that Red clones consistently maintained a higher fraction of tdTomato-positive cells compared with Mosaic clones (Fig. S4*A*), indicating stable maintenance of elevated MERVL reporter activity.

Representative bright field and fluorescence microscopy confirmed clear morphological and reporter-expression differences between Red and Mosaic R1 ESC clones (Fig. S4*B*). Red clones displayed widespread and homogeneous tdTomato signal across colonies, whereas mosaic clones showed patchy and fluctuating reporter expression within the same culture.

To assess the temporal stability of MERVL reporter activity, we performed live-cell imaging using the IncuCyte platform. Quantitative analysis of tdTomato-positive area relative to total colony area demonstrated that red R1 ESC clones maintained high and sustained MERVL reporter activity over time, while mosaic clones exhibited dynamic transitions between reporter-positive and reporter-negative states (Fig. S4*C*). These differences were evident across multiple independently derived clones, supporting the reproducibility of the red and mosaic phenotypes. Representative images extracted from the live-cell imaging time course further illustrated the stable persistence of tdTomato signal in Red R1 sMERVL ESCs, in contrast to the reduced and heterogeneous signal observed in Mosaic clones (Fig. S4, *D* and *E*).

Together, these experiments demonstrate that stable maintenance of MERVL-positive ESCs is reproducible across independent derivations, transfections, and clonal isolations in R1 ESCs. The emergence of both Red and Mosaic MERVL-positive populations in an independent ESC background supports the robustness of this system and indicates that stable MERVL-positive states are not restricted to a single ESC line or genetic context.

### Transcriptome profiling reveals a distinct 2C program in red sMERVL ESCs

**Figure 2:**
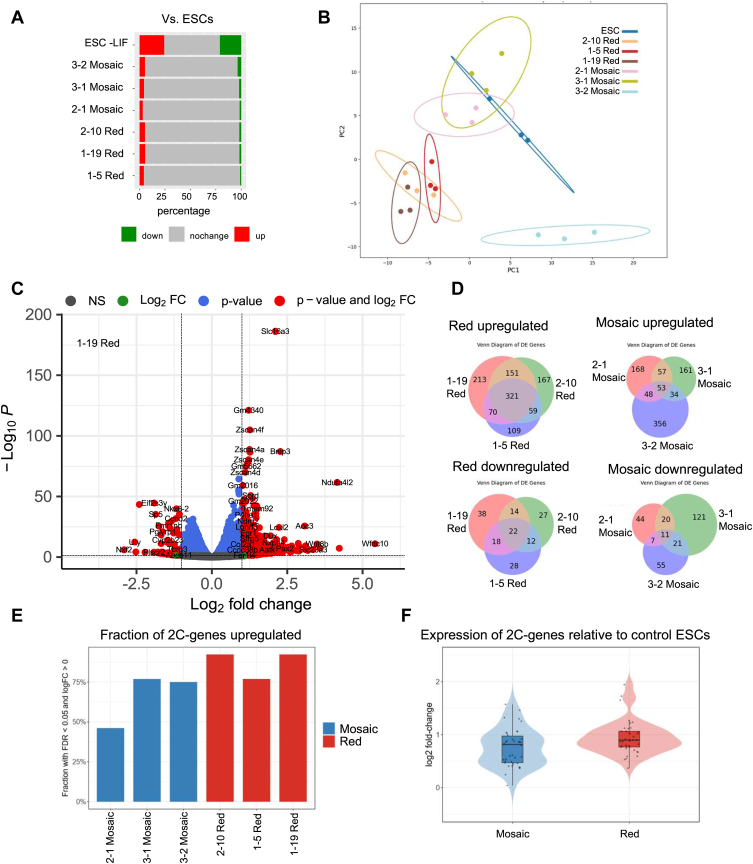
**Transcriptome analysis of sMERVL ESCs.***A*, differential expression analysis of MERVL-positive ESCs and differentiated ESCs compared with conventional ESCs, showing the number of upregulated and downregulated genes identified by edgeR. *B*, principal component analysis illustrating that red sMERVL ESCs clusters distinctly from conventional ESCs, whereas Mosaic sMERVL ESCs display greater variability and remain closer to conventional ESCs. *C*, volcano plot of differentially expressed genes between a representative *red* sMERVL ESCs line (1–19 *red*) and conventional ESCs. Each point represents a gene, plotted by log2 fold change (x-axis) and significance (-log10 *p*-value, y-axis). *Gray* points denote non-significant genes, *green points* represent significant fold changes, *blue points* indicate significant *p*-values, and *red points* highlight genes significant by both criteria. Elevated expression of transcription factors and other 2C-associated regulators is observed in *red* sMERVL ESCs. *D*, genes upregulated (*upper panels*) and downregulated (*lower panels*) in red (*left*) and *mosaic* (*right*) sMERVL ESCs. Venn diagrams show unique and shared upregulated genes across *red* and *mosaic* populations. *E* and *F*, quantification of activation of established 2C-associated gene programs across sMERVL ESCs. *E*, fraction of established 2C-associated genes significantly upregulated in *Red* and *Mosaic* sMERVL ESCs relative to conventional ESCs. *F*, distribution of log2 fold changes for established 2C-associated genes across *red* and *mosaic* sMERVL ESCs compared with conventional ESCs. ESC, embryonic stem cell; sMERVL ESC, stable maintenance of MERVL-positive ESC.

To characterize the expression profiles of red and mosaic MERVL-positive ESCs, and to understand the expression of 2C-genes, ERVs, and differentially expressed genes between red and mosaic MERVL-positive and conventional ESCs, we performed transcriptome analysis using RNA-Seq. Conventional ESCs (+LIF) and differentiated ESCs (-LIF) were used as controls (see Experimental procedure). edgeR differentially expressed (DE) analysis revealed differences in the number of upregulated and downregulated genes between Red and Mosaic sMERVL ESCs (Fig. 2*A* and Table S1). Principal component analysis (PCA) demonstrated that Red sMERVL ESCs are more distinct from conventional ESCs along the PC1 axis, which accounts for the majority of variance, compared to Mosaic sMERVL ESCs (Fig. 2*B*).

Volcano plots were used as an initial visualization of DE across clone-level comparisons, but they do not fully capture the behavior of canonical 2C markers across all samples (Figs. 2*C* and S5). Complementary MA plots confirmed consistent DE patterns across individual clones, supporting the robustness of these observations (fold-change and *p*-value; Fig. S6).

We identified 321 genes that were upregulated in Red sMERVL ESCs compared to conventional ESCs. In contrast, Mosaic sMERVL ESCs exhibited an upregulation of 53 genes relative to conventional ESCs. Additionally, there were 22 and 11 genes downregulated in Red and Mosaic sMERVL ESCs, respectively (Fig. 2*D*).

In a representative Red clone, genes such as Zscan4d, Dux, and other 2C-associated regulators were elevated relative to conventional ESCs (Figs. 2*C* and S5). However, targeted analysis of canonical 2C genes across all clone-level comparisons demonstrated that expression of individual Zscan family members is heterogeneous rather than uniformly detected in every clone (Fig. S7*A*). Zscan4-family genes, particularly Zscan4c and Zscan4d, were consistently detected in Red clones and variably detected in Mosaic clones, indicating clone-dependent differences in marker magnitude rather than absence of activation (Fig. S7*B*). Importantly, when assessed at the level of the broader transcriptional program rather than individual markers alone, 2C-associated genes showed reproducible enrichment across sMERVL ESCs, with Red clones exhibiting stronger and more consistent activation than Mosaic clones (Figs. 2, *E* and *F* and S7). These findings indicate that the 2C-like state in sMERVL ESCs is best interpreted as activation of a coordinated transcriptional program with superimposed clone-specific variability in individual marker genes.

Red sMERVL ESCs exhibited elevated expression of genes of the Zscan4 family (*e.g.* Zscan4c Zscan4d (7)), Dux (8, 9), Zim3, Wnt3, Wnt8b, and Trim56 (Figs. 2*C*, S5 and S6). Notably, Zscan4-family genes and Dux are key regulators of the 2C-like transcriptional state (7, 8, 9).

To further distinguish marker-level heterogeneity from program-level reproducibility, we quantified activation of core established 2C-associated genes across all clones. The established 2C gene set was curated from prior studies (4, 9), and includes canonical regulators (Dux, Zscan4 family), 2C-stage transcripts (Zfp352, Tcstv1/3, Tdpoz genes, Pramel6/7), and ERVL-associated genes (*e.g.*, Gm4340), together with additional 2C-enriched factors such as Sp110 and Dub1/2a. This analysis showed that the fraction of significantly upregulated 2C genes and the overall distribution of their expression changes were consistently higher in Red clones compared to Mosaic clones, although Mosaic clones also exhibited increased activation relative to conventional ESCs (Figs. 2, *E* and *F* and S7). Thus, despite variability in individual Zscan family members, Red sMERVL ESCs reproducibly exhibit the strongest 2C-like transcriptional signature at the gene-set level, with Mosaic clones representing a weaker and more heterogeneous intermediate state.

Gene ontology (GO) functional annotation of DE genes using clusterProfiler (27) allowed for the comparison of GO terms enriched in activated or suppressed genes in red and mosaic ESCs (Fig. S8). Specifically, pathways that were activated include transmembrane signaling receptor activity, positive regulation of cell adhesion, regulation of cell-cell adhesion, calcium ion binding, and signaling receptor activity, which were prominently activated in Red sMERVL ESCs. This suggests that Red sMERVL ESCs have heightened activities in cell adhesion, ion transport, and cell signaling.

Additional functional genomics analysis of regulated genes in Red sMERVL ESCs was conducted through Metascape (28). Enrichment networks were displayed using Cytoscape (29) to highlight GO clusters (Fig. S9*A*), and protein-protein interaction modules defined by MCODE (Fig. S9*B*). These networks revealed enrichment of transcriptional regulators in Red sMERVL ESCs. TRRUST analysis further identified targets of key transcription factors, including Stat3, as well as Ets1, Sp1, Etv2, Fos, Trp53, and Pparg, whose target gene expression was enriched relative to conventional ESCs (Fig. S10).

### Single-cell transcriptomics identifies distinct transcriptional states and cluster redistribution in sMERVL ESCs

To validate the transcriptional identity of MERVL-positive ESCs at single-cell resolution and to assess transcriptional heterogeneity, we performed FLEX-seq scRNA-seq on control ESCs and independent MERVL-positive lines (Red and Mosaic). All samples were processed, sequenced, and analyzed within a matched experimental and computational framework to minimize technical variability.

**Figure 3:**
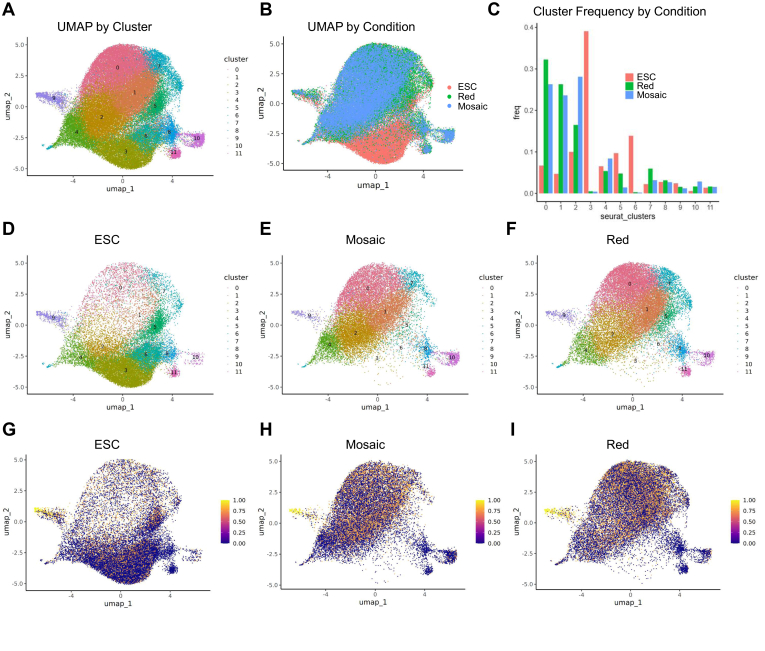
**Single-cell analysis reveals condition-dependent remodeling of transcriptional states in sMERVL ESCs.***A*, UMAP visualization of all single cells colored by unsupervised RNA-based clusters, derived from a non-integrated analysis to preserve condition-associated transcriptional structure. *B*, UMAP visualization of the same cells colored by condition (control ESCs, *mosaic* sMERVL ESCs, and *red* sMERVL ESCs), showing separation of control from MERVL-positive populations. *C*, cluster frequency distribution across conditions. *Bar plots* show the proportion of cells assigned to each cluster within each condition, highlighting condition-dependent shifts in transcriptional state abundance. *D*–*F*, UMAP projections showing individual conditions overlaid on the shared embedding: (*D*) control ESCs, (*E*) *mosaic* sMERVL ESCs, and (*F*) *red* sMERVL ESCs. These panels illustrate differential occupancy of the transcriptional manifold across conditions. *G*–*I*, overlay of 2C-associated gene expression across the UMAP (Macfarlan *et al.* 2C-gene set). Each point represents a single cell colored by the relative level of 2C-associated gene detection per cell. (*G*) control ESC, (*H*) *mosaic*, (*I*) *red*. 2C-associated transcriptional activity is broadly distributed across multiple clusters, with increased frequency of higher-scoring cells in *red* and *mosaic* MERVL-positive populations. ESC, embryonic stem cell; sMERVL ESC, stable maintenance of MERVL-positive ESC.

We analyzed the data using a non-integrated RNA-only workflow to preserve condition-associated transcriptional structure. This analysis revealed clear separation of control ESCs from Red and Mosaic MERVL-positive populations in UMAP space (Fig. 3, *A* and *B*). Quantification of cluster composition demonstrated reproducible redistribution of cells across transcriptional states (Fig. 3*C*), with multiple clusters enriched in MERVL-positive ESCs relative to control ESCs. Specifically, clusters 0, 1, 2, 7, and 10 were enriched in Mosaic and Red populations, whereas control ESCs showed greater enrichment of clusters 3, 5, and 6. A cluster enriched for 2C-associated features (cluster 9) was identified but did not exclusively define the MERVL-positive state. Red lines showed the strongest shifts, while Mosaic lines exhibited intermediate changes, indicating graded, condition-dependent remodeling of transcriptional states. Visualization of individual conditions confirmed differential occupancy of a shared transcriptional manifold rather than the emergence of a single dominant cluster (Fig. 3, *D–F*).

To relate these transcriptional states to previously defined 2C-like programs, we overlaid 2C-associated gene signatures (Macfarlan-derived 2C gene set) (Fig. 3, *G–I*). Canonical 2C-associated gene expression was detectable but did not define a spatially segregated population and was instead broadly distributed across multiple clusters in Mosaic and Red cells relative to control ESCs.

Visualization of per-cell 2C gene detection revealed a clear shift toward higher 2C-associated transcriptional activity across conditions. Mosaic and Red populations exhibited an increased frequency of cells with higher numbers of detected 2C-associated genes across the transcriptional landscape (Fig. 3, *G–I*). Consistent with this, cells with elevated 2C-associated gene detection were not confined to a single cluster but were distributed across multiple transcriptional states.

Together, these analyses indicate that 2C-like transcriptional activity is not confined to a discrete population but is instead represented as a distributed and enriched program across the transcriptional manifold. This pattern reflects the distribution of 2C-associated transcriptional features across multiple clusters rather than their restriction to a single state.

The RNA-only analysis reveals robust and reproducible restructuring of transcriptional states in MERVL-positive ESC populations. Control ESCs are clearly separated from Red and Mosaic conditions, with substantial shifts in cluster composition. These findings demonstrate that MERVL-positive ESCs occupy distinct transcriptional states relative to conventional ESCs, with 2C-like transcriptional activity distributed across a continuum of transcriptional states rather than confined to a spatially isolated cluster.

### Expression of endogenous retroelements in sMERVL ESCs

To further profile the expression of repetitive DNA elements in Red and Mosaic sMERVL ESCs, we used RepeatMasker annotations of repeat family members and repeat names, and performed edgeR analysis to identify DE elements relative to conventional ESCs. We observed several ERVL-class retrotransposons consistently upregulated in Red sMERVL ESCs, including MERVL-int, MT2_Mm, RLTR6_Mm, ORR1A3-int, and GSAT_MM, whereas Mosaic sMERVL ESCs showed significant upregulation of only RLTR13B2. These findings, illustrated in volcano plots (Fig. S11*A*) and heatmaps (Fig. S11, *B* and *C*) for both repeat families and ERVL elements (Table S2), confirm that Red sMERVL ESCs sustain elevated expression of MERVL-int and MT2_Mm. Importantly, the strong induction of RLTR6_Mm, ORR1A3-int, and GSAT_MM in Red sMERVL ESCs represents an expanded set of ERV-associated elements not previously linked to the 2C-like state. Conversely, several repetitive elements, including MER68-int, MLT1F1-int, Tigger9a, and U4, were consistently downregulated in Red sMERVL ESCs.

### Altered differentiation behavior of sMERVL ESCs revealed by embryoid body assays

2C-like cells have been associated with expanded developmental potential, including contributions to both embryonic and extraembryonic lineages. To assess whether sMERVL ESCs exhibit altered differentiation behavior, we performed embryoid body (EB) assays over a 12-day period to model early developmental transitions. Control ESCs and sMERVL ESCs were subjected to identical aggregation and differentiation conditions (LIF withdrawal) and analyzed at matched time points, ensuring direct comparability across lines.

**Figure 4:**
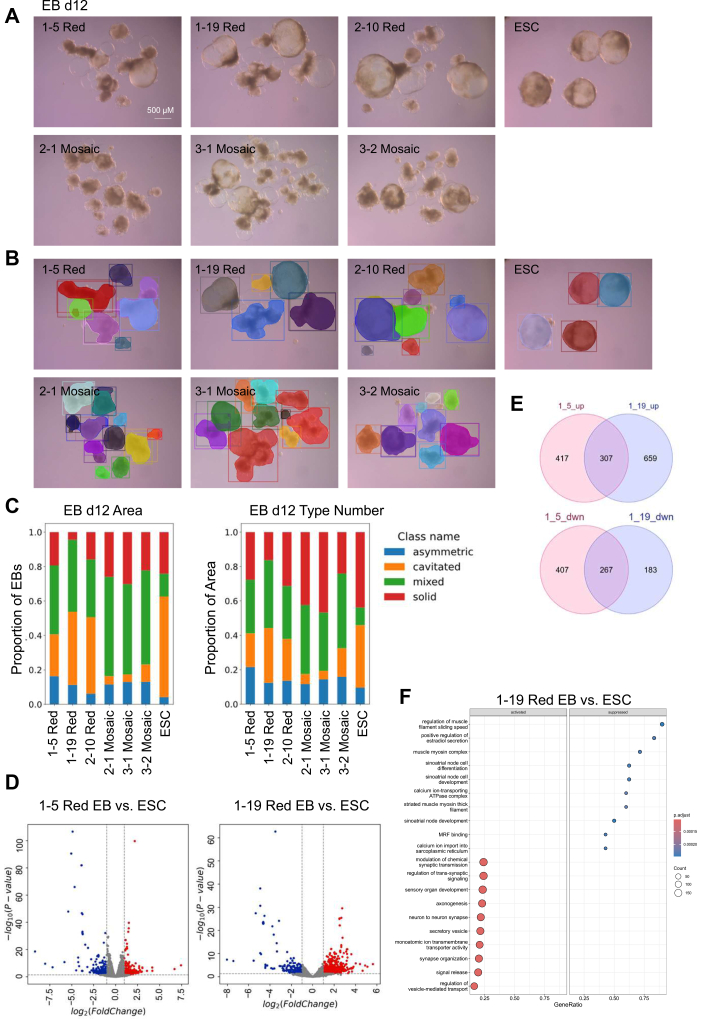
**Differentiation and transcriptome analysis of sMERVL ESCs.***A*, bright field microscopy of *Red* and *Mosaic* sMERVL ESCs, and conventional ESCs, differentiated into EBs without LIF for 12 days. *B*, Detectron2-based segmentation classified EBs as solid, asymmetric, cavitated, or mixed, and distributions were quantified by both EB number and EB area. *C*, image segmentation results from the Detectron2 model, shown as stacked bar plots depicting the distribution of EB morphologies by area and number. *D*, volcano plot of differentially expressed genes comparing day 12 EB-differentiated *red* sMERVL ESCs (clones 1–5 and 1–19) with conventional EBs. Genes are plotted by log2 fold change (x-axis) and significance (−log10 *p*-value, y-axis). *Gray* points represent nonsignificant genes, *green points* indicate significant fold changes, *blue points* denote significant *p*-values, and *red points* highlight genes significant by both criteria. (*E*) Venn diagram showing the overlap of upregulated and downregulated genes between *Red* sMERVL ESCs (clones 1–5 and 1–19) and conventional EB-differentiated ESCs. *F*, gene ontology enrichment analysis (clusterProfiler) of upregulated and downregulated genes in day 12 EB-differentiated *red* sMERVL ESCs (clone 1–19) compared with conventional EB-differentiated ESCs. EB, embryoid bodies; ESC, embryonic stem cell; sMERVL ESC, stable maintenance of MERVL-positive ESC.

Under these conditions, sMERVL ESCs generated EBs with reproducibly altered self-organization relative to control ESCs. Morphological analysis revealed increased heterogeneity in EB structure, reflected by greater variability in EB shapes and organization (Fig. 4*A*). These findings indicate that enrichment for sustained MERVL/2C-associated transcriptional programs is associated with altered differentiation behavior *in vitro*.

We note that EB assays provide an *in vitro* measure of differentiation behavior and do not constitute definitive evidence of expanded developmental potential *in vivo*. Accordingly, these results are interpreted as evidence of altered differentiation dynamics rather than expanded potency.

To quantify EB heterogeneity in an objective and scalable manner, we trained a Detectron2-based instance segmentation model to classify EBs into four operational morphology classes: solid (compact, noncavitated), asymmetric (nonround/polarized shape without a dominant lumen), cavitated (presence of a clear lumen/cavity), and mixed (EBs containing both solid and cavitated regions and/or heterogeneous organization). Morphology classes were used as quantitative readouts of EB self-organization and cavitation dynamics rather than direct lineage assignments. Segmentation highlighted distinctive features of Red and Mosaic sMERVL-positive-ESC-derived EBs compared to controls (Figs. 4*B* and S12, *A* and *B*). Notably, Red sMERVL-positive ESCs produced fewer solid EBs, and Red and Mosaic cells produced a higher proportion of mixed EBs (Fig. 4*C*). While morphology alone does not define lineage identity, these reproducible architecture shifts prompted us to test extraembryonic lineage programs directly using marker expression assays.

To probe differentiation trajectories at the transcriptional level, we performed RNA-seq after 12 days of EB differentiation in the absence of LIF. Red sMERVL ESC-derived EBs exhibited 307 upregulated and 267 downregulated genes relative to conventional ESC-derived EBs (Fig. 4, *D* and *E* and Table S3). GO enrichment analysis (clusterProfiler) identified biological processes enriched among upregulated genes, including pathways related to muscle and cardiac development, calcium handling, and contractile fiber organization (Figs. 4*F* and S13). Together, these findings demonstrate that sMERVL ESCs undergo altered and broadened differentiation programs, consistent with altered lineage potential.

### Molecular evidence of enhanced extraembryonic lineage programs during EB differentiation

To determine whether the altered EB morphologies observed in sMERVL ESCs reflect shifts in lineage specification, we performed independent EB differentiation experiments using control R1 ESCs and clonally derived Red and Mosaic sMERVL R1 lines. Bright-field imaging at day 12 revealed clear architectural differences between control and sMERVL-derived EBs (Fig. S14, *A* and *B*). Notably, both Red and Mosaic sMERVL lines exhibited a marked increase in solid, non-cavitated EBs, with a corresponding reduction in cavitated structures relative to control ESCs (Figs. S14*C* and S15, *A* and *B*), consistent with an altered differentiation trajectory.

To provide molecular validation of these morphology-based observations, cells were differentiated in the absence of LIF and harvested at days 0, 8, and 12 for quantitative RT-PCR analysis of lineage markers (Fig. S16).

We first examined canonical trophectoderm regulators. Cdx2, a master regulator of trophectoderm specification (30), was elevated at day 8 in multiple R1 Red and Mosaic MERVL-positive clones compared with control ESC-derived EBs. Elf5 (31), a key determinant of trophoblast lineage identity and downstream effector of Cdx2, was increased in most Red and Mosaic clones during differentiation. Gata3, which functions in trophoblast development and maintenance (32), was elevated in MERVL-positive clones at both day 8 and day 12. Gata2, another transcription factor implicated in trophoblast lineage regulation (33), was significantly increased in one clone and showed elevated trends in additional lines.

We next assessed primitive endoderm-associated markers. Sox17, a regulator of extraembryonic endoderm development (34, 35), was elevated in most Red and Mosaic MERVL-positive clones during differentiation. Foxa2, which contributes to endoderm specification (36), was similarly increased in several sMERVL lines (37). In contrast, Gata4 expression, although induced during EB differentiation as expected, was broadly comparable between control and sMERVL lines, suggesting selective enhancement of specific extraembryonic programs rather than generalized amplification of endoderm differentiation.

Together, these results demonstrate that sMERVL ESCs exhibit enhanced activation of both trophectoderm-associated and primitive endoderm-associated transcriptional programs during EB differentiation. The concordance between Detectron2-defined alterations in EB morphology (Fig. S12 and S15, *A* and *B*) and the induction of extraembryonic lineage regulators provides molecular support for an association between sustained MERVL activity and activation of extraembryonic lineage programs.

### Epigenome profiling of red and mosaic sMERVL ESCs

We next examined whether sMERVL ESCs are accompanied by distinctive chromatin landscapes. To do so, we analyzed H3K4me3 (38) (active promoters/transcription start sites (TSS) (39, 40, 41)) and H3K27ac (42) (active enhancers/SEs) by ChIP-seq.

**Figure 5:**
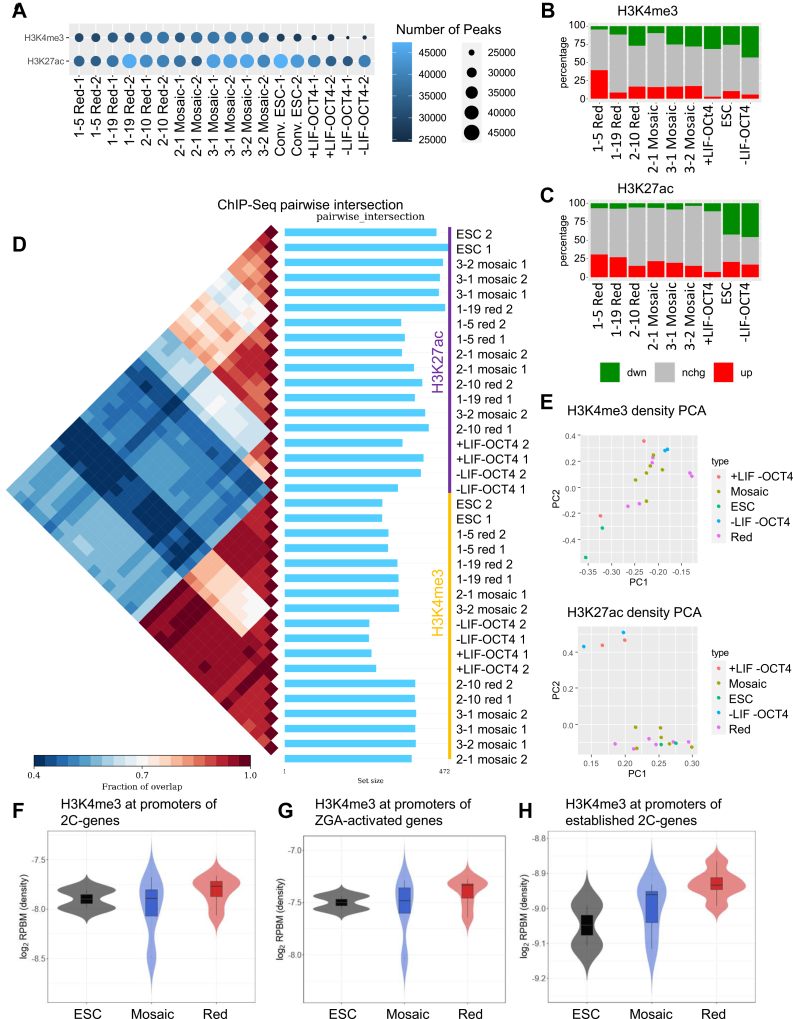
**Comparative epigenetic profiling of histone modifications in sMERVL ESCs across differentiation states.***A*, *bubble plot* showing the number of ChIP-enriched peaks for H3K4me3 and H3K27ac, with *bubble size* and *color* indicating peak counts. *B and C*, percentage change in (*B*) H3K4me3 and (*C*) H3K27ac levels across experimental conditions. *Red* indicates increased peaks and *green* indicates decreased peaks, as determined by SICER-compare analysis of multiple samples. These comparisons reveal differential epigenetic regulation in *red* and *mosai*c sMERVL ESCs relative to conventional ESCs. *D*, pairwise comparison of ChIP-enriched peak overlaps performed using Intervene. *E*, principal component analysis of normalized H3K4me3 and H3K27ac tag densities. *F* and *H*, promoter-associated H3K4me3 density at canonical 2C/ZGA gene sets. *Violin* plots show log_2_ RPBM signal density at promoter regions for (*F*) Macfarlan-defined 2C genes, (*G*) MERVL/ZGA-associated chimeric transcripts, and (*H*) curated established 2C gene sets. ESC, embryonic stem cell; sMERVL ESC, stable maintenance of MERVL-positive ESC.

We first quantified global peak numbers across Red and Mosaic sMERVL ESCs, conventional ESCs, and differentiated ESCs. H3K4me3 peak counts were heterogeneous but consistently elevated in Red and Mosaic sMERVL ESCs compared to ESCs and differentiated cells, ranging from ∼29,857-37,727 in Red clones (Fig. 5*A*, top). H3K27ac peaks also increased, ranging from ∼33,663-39,794 in Red sMERVL ESCs (Fig. 5*A*, bottom). Mosaic clones tended to show slightly higher counts, consistent with a more variable transcriptional landscape. In contrast, differentiated ESCs showed reduced H3K4me3, highlighting the loss of promoter activity upon exit from pluripotency.

To systematically compare signal dynamics, we applied SICER-compare (FDR < 0.001, FC > 1.5) (43). Red and Mosaic sMERVL ESCs showed significant increases in H3K4me3 and H3K27ac peaks relative to ESCs, whereas -LIF/-OCT4 differentiation caused pronounced losses (Fig. 5*B*). For example, the “1–5 Red” clone exhibited increased H3K4me3, while -LIF/-OCT4 cells had decreased H3K4me3. Two red clones also showed elevated H3K27ac compared to ESCs (Fig. 5*C*), suggesting enhancer hyperactivation.

Because peak counts and percent-change summaries can be sensitive to clone-to-clone heterogeneity, we evaluated clonal structure in the H3K27ac datasets. Unsupervised PCA of H3K27ac profiles separates sMERVL ESCs from ESC controls and shows that Red clones exhibit a larger global shift than Mosaic clones, consistent with tdTomato intensity capturing a distinct epigenomic state. (Fig. S17*A*).

To quantify differential H3K27ac changes at the clone level, we used SICER differential output for each clone relative to the ESC control comparator and counted the number of differential increased regions (and, separately, all differential regions). While within-group variability is evident, Red clones consistently show a greater number of increased H3K27ac regions than Mosaic clones. (Fig. S17*B*).

Because global peak counts provide only a coarse summary, we next evaluated the functional context of differential histone-mark changes. Differential regions identified by SICER-compare (increased and decreased sets; Fig. 5, *B* and *C*) were analyzed using GREAT (44) to associate regions with nearby genes and to identify enriched GO Biological Process terms. GREAT summaries for H3K4me3 and H3K27ac are shown in new supplementary figures (Figs. S18 and S19), providing pathway-level interpretation of chromatin gains *versus* losses in sMERVL ESCs.

Peak intersections using Intervene (45) showed that Red and Mosaic clones shared large fractions of H3K4me3/H3K27ac peaks, clustering more closely with one another than with ESCs or differentiated states (Fig. 5*D*). PCA of H3K27ac densities across merged peaks clearly separated sMERVL ESCs from ESCs and differentiated cells (Fig. 5*E*), indicating that enhancer acetylation better discriminates the MERVL-positive state than H3K4me3.

Metagene profiles of H3K4me3 and H3K27ac across TSS to polyA regions further illustrated globally elevated activation marks in sMERVL ESCs (Fig. S20). HOMER (46) annotation confirmed enrichment of both marks in promoters, introns, and intergenic regions (Fig. S21, *A* and *B*). deepTools (47) k-means clustering identified subsets of genes with broad H3K4me3 domains and distinct H3K27ac enrichment at highly expressed loci (Fig. S21, *C* and *D*). Density comparisons against ESCs confirmed broad promoter and enhancer activation in Red and Mosaic sMERVL ESCs (Fig. S21, *E* and *F*).

Building on these global analyses of chromatin activation, we sought to determine whether these changes are preferentially associated with genes defining the 2C-like state. To address this, we performed gene-centric analyses of promoter-associated H3K4me3 signal across canonical 2C/ZGA gene sets (Fig. 5, *F*–*H*). Across these gene sets, we observed a consistent increase in promoter H3K4me3 signal from ESCs to Mosaic and Red sMERVL ESCs, indicating progressive promoter activation associated with the sMERVL state.

Notably, Mosaic sMERVL ESCs displayed a broader distribution of promoter H3K4me3 signal, with increased variance and a subset of loci exhibiting strong activation, consistent with a heterogeneous or transitional chromatin state. In contrast, Red sMERVL ESCs exhibited a more compact and uniformly elevated distribution, indicating coordinated and stabilized promoter activation across 2C-associated genes.

This pattern was reproducible across multiple independent gene sets, including Macfarlan-defined 2C genes (Fig. 5*F*), ZGA/MERVL-associated transcripts (Fig. 5*G*), and curated established 2C gene sets (Fig. 5*H*), demonstrating that promoter activation broadly targets genes defining the 2C-like transcriptional program.

### Enhancer remodeling in sMERVL ESCs: ERV-proximal activity and super enhancers

**Figure 6:**
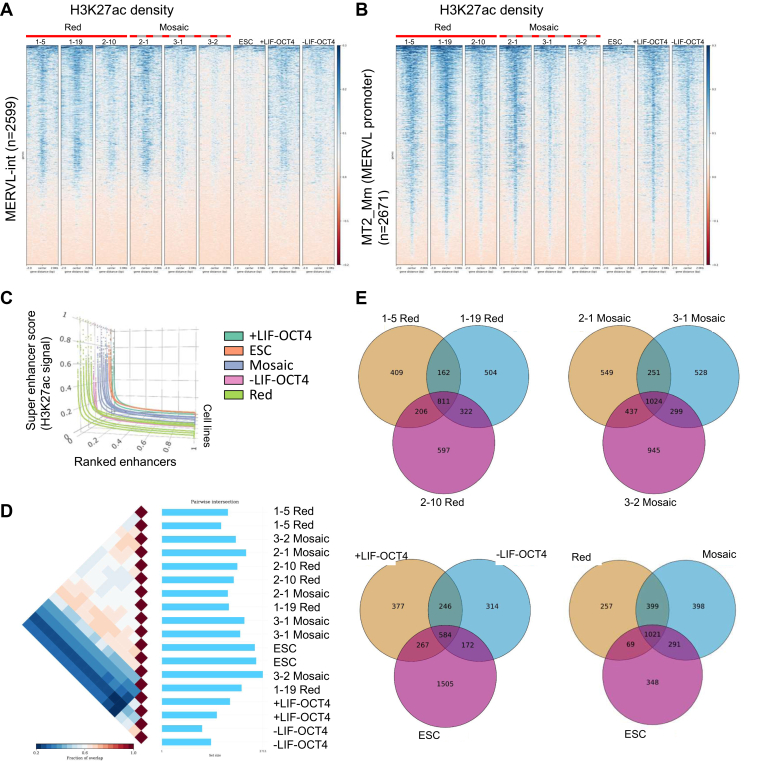
**Differential enrichment of H3K27ac and SE analysis of sMERVL ESCs.***A* and *B*, distribution of H3K27ac signals around (*A*) MERVL-int and (*B*) MT2_Mm (MERVL promoter) repeat elements, generated using deepTools. *C*, saturation curve of H3K27ac densities in sMERVL ESCs and ESCs, showing the distribution of typical enhancers and SE ranked by H3K27ac density. Normalized H3K27ac ChIP-seq signals are plotted across enhancer regions. SE were identified using HOMER (see Experimental procedures) and defined as regions with a slope >1. *D*, pairwise comparison of SE overlaps across MERVL-positive cells and ESCs under different culture conditions, performed with Intervene. *E*, venn diagram comparing shared SEs in *red* and *mosaic* clones, and ESCs, showing that the shared *red-mosaic* SE component contains both ESC-conserved and sMERVL-associated subsets. ESC, embryonic stem cell; SE, super enhancers; sMERVL ESC, stable maintenance of MERVL-positive ESC.

Given the central role of ERVs in 2C biology, we next asked whether enhancer activation is associated with MERVL loci. deepTools density analysis revealed that both MERVL-int and MT2_Mm were strongly enriched for H3K27ac in Red clones, but only weakly marked in Mosaic clones, or differentiated cells (Fig. 6, *A* and *B*). These findings indicate that ERV-associated transcriptional activity in Red clones is accompanied by proximal enhancer acetylation.

To extend this view, we identified SEs by ranking stitched H3K27ac peaks (HOMER (46) -superSlope −1000; merge window 12.5 kb). Red sMERVL ESCs displayed high-intensity SEs distinct from Mosaic sMERVL ESCs, control ESCs, and differentiated cells (Fig. 6*C*).

Overlap analysis of H3K27ac-defined SEs revealed largely condition-specific SE landscapes across sMERVL cells and ESC controls, with limited sharing between groups (Fig. 6*D*). Despite this, a subset of SEs was shared between Red and Mosaic sMERVL ESCs (Fig. 6*E*). To determine whether these shared SEs are unique to sMERVL ESCs or conserved from ESCs, we extended the analysis to include SEs identified in conventional ESCs and performed intersections using reproducible SE sets defined within each condition across biological replicates. This analysis identified two classes of shared SEs: an ESC-conserved subset present in control ESCs and both sMERVL populations, and a smaller subset shared between Red and Mosaic cells but not detected in ESCs, representing candidate sMERVL-associated SEs (Fig. 6*E*). Thus, the shared SE component of Red and Mosaic sMERVL ESCs consists primarily of ESC-conserved elements, with a smaller fraction potentially specific to the sMERVL state. Fingerprinting analysis of H3K4me3, H3K27ac, and Input confirmed differential coverage across samples (Fig. S22, *A*–*C*).

Together, these results indicate that MERVL-positive ESCs exhibit two layers of enhancer remodeling: (i) ERV-proximal H3K27ac enrichment associated with MERVL loci, and (ii) condition-specific SE landscapes with a partially shared regulatory component between Red and Mosaic states.

### Chromatin state modeling reveals ERV integration into H3K27ac-rich states

To provide an intuitive, mark-specific view of ERV-proximal enhancer activation, we focused on direct H3K27ac signal enrichment at MERVL-associated repeats. H3K27ac is strongly enriched at MERVL-int and MT2Mm in Red sMERVL ESCs relative to Mosaic sMERVL ESCs and controls (Fig. 6, *A* and *B*), consistent with enhancer activation at ERV loci. A ChromHMM-based two-mark summary is provided as a supplementary analysis (Fig. S23*A*) and is interpreted conservatively given the limited number of marks.

Neighborhood enrichment revealed distinct distributions of MERVL-int: in ESCs, MERVL-int localized primarily to State 4 (H3K4me3-only), whereas in Red sMERVL ESCs it shifted into States 1 and 3 (H3K27ac-enriched), with moderate retention in State 4 (Fig. S23*B*). Mosaic sMERVL ESCs exhibited mixed enrichment across States 1/3/4, while differentiated cells favored States 3 and 4. Thus, Red sMERVL ESCs reposition MERVL into H3K27ac-rich states, aligning ERV transcription with enhancer activity.

ChromHMM applied to repeat classes showed that ERV/LTRs were preferentially enriched in Red and Mosaic sMERVL ESCs compared to ESCs or differentiated cells (Fig. S23*C*). Pearson correlations of ChromHMM enrichments clustered Red and Mosaic sMERVL ESCs together, apart from ESCs and differentiated cells (Fig. S23*D*). Analysis of RNA repeats supported this separation (Fig. S24), with low-complexity and satellite repeats showing distinct profiles. Notably, srpRNA repeats correlated specifically between red sMERVL ESCs and one mosaic clone, pointing to clone-specific features.

At the DNA repeat family level, ERVs localized predominantly to States 1 and 3 in Red and Mosaic sMERVL ESCs, while conventional and differentiated ESCs favored State 4 (Fig. S23*E*). Other repeat families (ERV1, LTR, RNA repeats) exhibited similar patterns (Fig. S25). Pearson correlations further confirmed stronger similarity between Red and Mosaic sMERVL ESCs than with ESCs or differentiated states (Fig. S23*F*).

These analyses establish that sMERVL ESCs are characterized by the redistribution of ERVs into H3K27ac-rich chromatin states, coupled with enhancer remodeling and SE establishment, which together stabilize the 2C-like transcriptional program.

## Discussion

In this study, we demonstrate that ESCs can be stably maintained in a MERVL-positive state, defined by persistent activation of MERVL elements and sustained expression of 2C-specific genes. Unlike the sporadic and heterogeneous bursts of MERVL activity in conventional ESCs, the MERVL-positive ESCs described here exhibit sustained MERVL activity over several passages, as confirmed by live imaging and flow cytometry. This persistence is accompanied by induction of canonical 2C regulators such as Zscan4 and Dux, transcription factors that are critical for early embryonic transcriptional reprogramming. Dux, a pioneer transcription factor that drives ZGA and activates MERVL transcription (8, 9), was strongly upregulated in Red sMERVL ESCs. Zscan4, expressed in 2C-stage blastomeres and transiently in ESCs (7), was also robustly induced. Although only ∼5% of ESCs typically express Zscan4 at any one time, nearly all cells activate it over successive passages (48). Our results suggest that stabilizing Dux- and Zscan4-positive cells requires an accompanying chromatin environment that permits persistent MERVL activity.

Epigenomic profiling further underscored that sMERVL ESCs possess distinct chromatin states and enhancer architectures, including elevated promoter- and enhancer-associated marks (Fig. 5), the redistribution of H3K27ac to MERVL elements (Fig. S23), and the formation of SE clusters (Fig. 6). These features distinguish Red and Mosaic sMERVL ESCs from conventional ESCs and differentiated states, supporting the idea that stabilization of the MERVL-positive state involves broad remodeling of promoter and enhancer landscapes. Our analyses of MERVL-associated enhancer acetylation provide a mechanistic link between ERV transcriptional activity and the persistence of the 2C program.

A key finding is the identification of two distinct MERVL-positive subpopulations. Mosaic clones displayed heterogeneous and fluctuating reporter activity, while Red clones exhibited uniformly high reporter expression and greater stability. These divergent expression patterns were mirrored at the transcriptomic and epigenomic levels: Red sMERVL ESCs expressed a broader repertoire of 2C-specific genes and ERVL retrotransposons, whereas Mosaic sMERVL ESCs appeared to represent an intermediate or less stable state. This heterogeneity highlights that even within MERVL-positive cells, distinct pathways may govern the acquisition and maintenance of the 2C-like state. Together, these observations support a model in which Red and Mosaic populations represent metastable, clonally propagating states within the ESC landscape that can be maintained over experimentally relevant time frames, rather than fixed or terminal cell identities.

An important insight from the transcriptomic analysis is that canonical 2C markers are not uniformly expressed across all clones. In particular, expression of individual Zscan family members varies in magnitude between Red and Mosaic lines and across clone-level comparisons. We explicitly acknowledge this heterogeneity, as Zscan genes are widely used markers of the 2C-like state. Focused reanalysis indicates that this variability reflects clone-dependent fluctuations in individual marker expression rather than absence of a 2C-like transcriptional program. When evaluated at the gene-set level, canonical 2C-associated genes show consistent activation, with Red clones exhibiting the strongest and most reproducible signal, and Mosaic clones displaying a weaker and more heterogeneous intermediate pattern. These findings underscore the importance of distinguishing between marker-level variability and program-level reproducibility, and support interpretation of sMERVL ESCs as a spectrum of 2C-like states, with Red cells representing the most robust manifestation of this transcriptional program.

Our repeat expression analyses revealed that Red sMERVL ESCs consistently upregulate multiple ERVL family retrotransposons (MERVL-int, MT2_Mm, RLTR6_Mm, ORR1A3-int, GSAT_MM), while Mosaic sMERVL ESCs uniquely upregulated RLTR13B2. Our analyses revealed correlative upregulation of RLTR6_Mm, ORR1A3-int, and GSAT_MM in Red sMERVL ESCs, suggesting these elements may serve as additional markers of the stable 2C-like state. Prior studies have shown that MERVL elements function as alternative promoters for early embryonic genes and modulate chromatin accessibility (4, 49, 50). Our data build on these findings by showing that different ERVL subfamilies contribute variably to 2C identity depending on cellular context, suggesting specialized regulatory roles for distinct retrotransposons in stabilizing or destabilizing totipotency.

The connection between ERV activity, transcription factor networks, and chromatin remodeling revealed here provides new insight into how ESCs toggle between pluripotency and totipotency. Evolutionary co-option of retrotransposons into host regulatory networks may explain why MERVL and related ERVs are central to ZGA and to the 2C program. By capturing a stable MERVL-positive state, we provide a tractable *in vitro* system to interrogate how ERV-driven transcription shapes developmental potential and to test whether modulation of enhancer states can shift cell fate.

Functionally, sMERVL ESCs demonstrated altered differentiation outcomes in EB assays and these phenotypes were supported by increased expression of extraembryonic lineage regulators during EB differentiation. These results support the idea that stabilization of the 2C program directly influences lineage trajectories, reinforcing the link between ERV activation, chromatin remodeling, and altered differentiation behavior. Conventional ESC cultures, in which MERVL + cells are rare and transient, are limited for such studies. By contrast, sMERVL ESCs provide an enriched and reproducible model for dissecting totipotent-like features.

Importantly, the altered morphology of sMERVL-induced EBs is accompanied by transcriptional activation of both trophectoderm and primitive endoderm regulators. Cdx2 and Elf5 define the trophoblast lineage hierarchy (30, 31), while Gata3 and Gata2 function in trophoblast development and maintenance (32, 33). In parallel, Sox17 and Foxa2 regulate extraembryonic endoderm specification (34, 35, 36), whereas Gata4 is broadly induced during endoderm differentiation. The coordinated induction of these extraembryonic programs suggests that stable MERVL-positive ESCs possess increased differentiation plasticity *in vitro*. These findings align with prior reports demonstrating that 2C-like cells exhibit increased contribution to extraembryonic lineages.

While the differentiation phenotypes observed in EB assays support altered lineage behavior and increased plasticity, these assays do not provide definitive evidence of expanded developmental potency *in vivo*. Future studies using *in vivo* functional assays, such as chimera formation, will be required to rigorously assess developmental potential.

Taken together, our findings establish that the stable maintenance of 2C-like cells is achievable *in vitro* and that this state is defined by coordinated regulation of transcription factors, retrotransposons, and chromatin states. By elucidating the molecular and epigenetic features of sMERVL ESCs, our study extends current understanding of how ESCs access the 2C-like state and demonstrates that this state can be captured and sustained. These results not only illuminate fundamental principles of early embryonic gene regulation but also provide a foundation for future investigations into ERV biology, reprogramming, and regenerative medicine. In summary, the establishment of stable 2C-like cells offers a robust new model for investigating the interplay between retroelements, chromatin organization, and developmental potential in mammalian cells.

## Experimental procedures

### ESC cell culture

ESCs were cultured according to previously established methods with some alterations (51, 52). ZHBTc4 ESCs (OCT4-regulatable (53)) and R1 ESCs were maintained feeder-free on gelatin-coated tissue culture dishes in ESC medium (DMEM, 15% FBS, LIF/ESGRO) at 37 °C and 5% CO_2_, supplemented with 1.5 μm CHIR99021 (GSK3 inhibitor). ZHBTc4 ESCs are widely employed to study both self-renewal and differentiation, as they retain pluripotency under maintenance conditions yet can be driven to differentiate through OCT4 downregulation. Unless otherwise indicated, “conventional ESCs” refers to cultures maintained in LIF without doxycycline. To downregulate OCT4, ZHBTc4 ESCs were cultured in the same medium supplemented with doxycycline (2 μg/ml). R1 ESCs were maintained under feeder-free, LIF-supported conditions and were used for independent derivation of MERVL-positive lines and EB differentiation experiments as described in the Results. For differentiation experiments, ESCs were cultured in the absence of LIF (± doxycycline as indicated). Cells were routinely passaged after PBS washing and trypsin dissociation using serological pipettes (sc-200279, sc-200281).

### Generation and isolation of MERVL reporter-positive ESCs

To isolate MERVL/2C-like cells, we used a MERVL promoter-driven 2C::tdTomato reporter plasmid (4) (Addgene #40281) and modified it to include a HisD selectable cassette (from pENTR-R2L3-cGR-ID; Addgene #139541). Both ZHBTc4 and R1 ESCs were transfected with the modified reporter using Lipofectamine 2000 and selected with histidinol (800 μm−1.6 mM). After selection, resistant populations were expanded and tdTomato-positive cells were prospectively isolated by FACS and then clonally expanded to establish independent reporter-positive ESC lines. Throughout the manuscript, “stable MERVL-positive (sMERVL)” refers to clonally derived tdTomato-positive lines that maintain a substantial tdTomato-positive fraction upon expansion. ZHBTc4 ESCs were used for OCT4-regulatable experiments and initial reporter characterization (Fig. 1*A*), whereas R1 ESCs were independently transfected and used to validate reproducibility of stable MERVL-positive line derivation across genetic backgrounds.

### Flow cytometry analysis

For flow cytometry, ESCs were washed with 1 × PBS, dissociated with trypsin to single cells, and resuspended at 1 × 10^6^ cells/ml in FACS buffer (PBS + 1% FBS). tdTomato gates were defined using parental reporter-negative ESCs and/or a tdTomato-positive control population (as indicated), and applied consistently across experiments. Dead cells were excluded by DAPI staining, and live reporter-positive cells were defined as tdTomato+/DAPI−. Flow cytometry data were collected on a BD LSR II and analyzed using FlowJo software (https://www.flowjo.com/). Experiments were performed with three biological replicates.

### Quantitative RT-PCR

Control R1 ESCs and independently derived Red and Mosaic MERVL-positive R1 clones were subjected to EB differentiation in the absence of LIF. Cells were harvested at day 0, day 8, and day 12 of differentiation. Total RNA was isolated using the Qiagen RNeasy Mini Kit according to the manufacturer’s instructions. RNA concentration and purity were assessed spectrophotometrically prior to reverse transcription using a Nanodrop. cDNA synthesis was performed using the High-Capacity cDNA Reverse Transcription Kit (Applied Biosystems) with random primers, following the manufacturer’s protocol. Quantitative real-time PCR was conducted using a SYBR Green 2X Master Mix on a QS5 real-time PCR system. Relative gene expression levels were normalized to 18S rRNA and calculated using the ΔΔCt method.

### Real-time imaging

The 2C::tdTomato ES cells were imaged every 30 min over a period of 72 h using the Sartorius IncuCyte SX five live-cell imaging system. The analysis focused on assessing the tdTomato area relative to the phase area (which corresponds to the ES cell colony size) and the total tdTomato area, measured in μm^2^/image. Representative images are displayed in Figure 1, *I* and *J*

### Training a Detectron2 model for precise segmentation of embryoid body images

#### Data preparation

We applied a machine learning approach, using a Detectron2 model (https://github.com/facebookresearch/detectron2), to quantify variations and to segment EBs into several morphological categories: solid, asymmetric, cavitated, and mixed. This approach was used to reduce subjective manual scoring and to enable consistent quantification of EB morphology distributions across conditions. For each sample, morphology composition was summarized both by EB number and by EB area derived from segmentation masks. The dataset was manually annotated using annotation software capable of exporting annotations in COCO JSON format (https://www.makesense.ai/). We used the polygon tool for object annotation and exported the annotations as a single file in COCO JSON format.

#### Model training

We installed Detectron2 by cloning the repository from GitHub and setting up the environment with necessary dependencies. The installation process involved the following steps.

#### Model configuration and training

We configured the Detectron2 model for instance segmentation using the DefaultPredictor class from Detectron2. The configuration steps included setting up the model configuration, specifying the dataset, and defining the training parameters.

#### Model testing and evaluation

We tested the trained model on a set of validation images. The images were preprocessed and fed into the model for prediction. The output masks were visualized to assess the model’s performance in segmenting the EBs.

### ChIP-seq analysis

ChIP-Seq experiments followed previously described methods with some modifications (54, 55). We used the rabbit monoclonal antibody H3K4me3 (17–614) from Millipore and the rabbit polyclonal H3K27ac (ab4729) from Abcam. Briefly, ESCs were crosslinked using 1% formaldehyde for 10 min at 37 °C. The fixed cells were then frozen at −80 °C. Later, these cell pellets were thawed and sonicated. For ChIP assays, extracts from four million cells were used along with 4 μg of antibody. The ChIP-enriched DNA was processed using the NEBNext Ultra II End Repair/dA-Tailing module, followed by ligation of Illumina adapters. PCR was conducted with the Phusion 2X High Fidelity PCR master mix. The prepared ChIP libraries were then sequenced using Illumina HiSeq platforms following the manufacturer's protocols. Sequence reads were aligned to the mouse genome (GRCm37) *via* bowtie2 (56) using default settings. For data processing, previously outlined C++ programs (57) were utilized for: converting SAM-formatted files to BED6 format from bowtie2 (Sam2Bed6_Bowtie2), removing redundant reads from a BED6 file (RemoveRedundantReads), and converting a BED6 files to a BEDGraph file (GenerateRPBMBasedSummary).

ChIP-Seq enriched regions (peaks) were detected by comparing them against control Input DNA using SICER (43). The settings applied were a window size of 200 bp, a gap size of 400 bp, and an FDR of 0.001. For analyzing multiple samples, the SICER-compare function was used with criteria of FDR< 0.001 and a fold-change >1.5. Normalization of ChIP-Seq libraries was carried out based on the size of the library, employing the RPBM metric (reads per base per million reads) to measure densities in genomic areas from ChIP-Seq datasets. The analysis included two biological replicates. Visualization of the normalized ChIP data was achieved through the UCSC genome browser.

### RNA-seq analysis

Poly-A mRNA was extracted using the NEBNext Ultra II RNA Library Prep Kit for Illumina. RNA-Seq libraries were sequenced on an Illumina system in accordance with the manufacturer’s instructions. Sequence reads were aligned to the mouse genome (GRCm37) using bowtie2 (56) with default settings. The RPKM metric (reads per kilobase of exon model per million reads) (58) was used to measure the mRNA expression levels of genes from RNA-Seq data. Genes showing DE were determined using edgeR, applying criteria of an FDR less than 0.001 and a fold change greater than 1.5) (59). RNA-Seq analysis was conducted with three biological replicates.

To further evaluate clone-level heterogeneity of canonical 2C markers, additional targeted analyses were performed on DE results from control-versus-clone comparisons. Established 2C-associated genes and Zscan4-family genes were examined separately to distinguish variability in individual markers from coordinated activation of the broader 2C-like transcriptional program. For each clone, we quantified mean log2 fold-change, the fraction of significantly upregulated genes within the established 2C gene set, and the distribution of log2 fold-changes across the gene set.

### FLEX-seq single-cell RNA sequencing

Single-cell RNA sequencing was performed using the 10x Genomics Chromium Single Cell Gene Expression Flex platform with the Chromium Mouse Transcriptome Probe Set v1.0.1 (mm10–2020-A). Libraries were generated according to the manufacturer’s protocol and sequenced on an Illumina platform. A total of 1.45 billion reads were obtained for the pooled Flex library. The mean reads per cell was 18,003, with a median of 10,145 UMI counts and 4166 genes detected per cell. Across samples, 10,243 to 19,241 cells were recovered per replicate, yielding robust coverage of transcriptional heterogeneity. Reads mapped efficiently to the probe set, with 98.29 percent of reads mapped to the probe set and 97.31 percent confidently mapped in cells. Sequencing saturation was 9.58 percent. In total, 16,425 genes were detected across the dataset. Raw base call files were demultiplexed and converted to FASTQ format using Cell Ranger (v8.0.1) (https://www.10xgenomics.com/support/software/cell-ranger/latest). Alignment and gene counting were performed against the mm10 reference genome (refdata-cellranger-mm10–1.2.0) using the Cell Ranger multi workflow with probe barcode demultiplexing enabled. The create-bam option was specified to retain aligned BAM files for downstream analyses. Six biological samples were analyzed across three experimental conditions, each with two independent replicates: control ESCs (ZHBTc4 ESC), red 2c::tdTomato-positive ESC clones, and mosaic 2c::tdTomato-positive ESC clones. Gene-barcode count matrices generated by Cell Ranger were used for downstream analysis.

### Single-cell data processing and RNA-only clustering analysis

Downstream analyses were conducted in R using Seurat v5. Cells were filtered to remove low-quality profiles, excluding cells with low gene detection and cells with greater than 20 percent mitochondrial transcripts, as well as cells with abnormally high gene counts indicative of potential doublets. After quality control filtering, 68,135 high-quality cells were retained for analysis. Gene expression values were normalized using SCTransform, and highly variable genes were identified for dimensionality reduction. Principal component analysis was performed, and the top principal components were used to construct a shared nearest neighbor graph. Clustering was performed using the Louvain algorithm on the RNA assay. Uniform manifold approximation and projection was computed on the RNA assay using principal components. To preserve condition-associated transcriptional structure, clustering and visualization were performed using a non-integrated RNA-only workflow rather than integrated embeddings.

### Cluster frequency analysis

To assess condition-dependent changes in cellular composition, cluster frequencies were calculated for each biological replicate. The proportion of cells assigned to each cluster was determined by dividing the number of cells in that cluster by the total number of cells in the replicate.

### Single-cell 2C gene program activity analysis

2C-associated gene program activity was evaluated using curated gene sets derived from previously published 2C-gene signatures (4). For each cell, gene set activity was quantified as the average normalized expression of genes within each set. In parallel, the number of detected genes per set was calculated to assess per-cell gene activation. Gene set activity was visualized across UMAP embeddings (Fig. 3, *G*–*I*). These analyses were used to assess condition-dependent enrichment and distribution of 2C-associated transcriptional activity across the transcriptional manifold.

### Chromatin state learning

Chromatin states in MERVL-positive ESCs were determined using ChromHMM v1.2 (60), which utilizes a multivariate Hidden Markov Model approach. The ChromHMM model was configured by merging data on histone modifications (ChIP-enriched peaks, refer to ChIP-Seq methods described previously) specifically for H3K4me3 and H3K27ac. For each ChIP-Seq dataset, peaks were evaluated in 200 bp bin intervals across the genome. These bins were classified into two categories: 1 for peak enrichment and 0 for no enrichment. A four-state model was chosen because it effectively identified key combinatorial patterns of histone modifications across the genome.

### Chromatin state annotations

The four chromatin states in the ESC epigenome were annotated using CpG islands from the UCSC Genome Browser, and genic features such as TSS, transcription end sites, genes, exons, and introns were incorporated into ChromHMM using ENCODE annotations, following previously described methods.

### SE analysis

SEs marked by H3K27ac was detected using HOMER (46). Initially, all enhancers were evaluated using the findPeaks function of HOMER and plotted with the addition of the "-superSlope −1000" parameter. H3K27ac peaks within 12.5 kb of each other were consolidated. The signal of each SE area was derived by deducting the count of normalized input reads from the count of normalized reads. These areas were then sorted, scaled relative to the highest value, and the quantity of typical enhancer regions was calculated. SE were designated as areas exceeding a slope of 1 (slope >1).

### Metascape network analysis

Metascape (28) was used to perform GO functional annotation and to provide functional insights into genes. This included GO enrichment analysis covering biological processes, canonical pathways, and WikiPathways results. Additionally, protein network analyses were carried out using Metascape.

### Heatmaps

The "computeMatrix" and "plotHeatmap" functions in the DeepTools (47) software suite were used to generate heatmaps and profile plots for histone marks and transcription factor binding from ChIP-seq datasets. These visualizations focused on regions extending from -3kb to +3 kb around the peak centers.

## Ethics approval and consent to participate

Not applicable.

## Consent for publication

All authors have read and approved the final version of this manuscript.

## Statistics and reproducibility

We generated biological duplicate H3K4me3 and H3K27ac ChIP-Seq datasets. We also generated biological triplicate RNA-Seq datasets, and biological duplicate FLEX-Seq scRNA-Seq datasets.

### Data availability

The sequencing data from this study have been submitted to the NCBI Gene Expression Omnibus under accession no. GSE275030.

## Supporting information

This article contains supporting information.

## Conflict of interest

The authors declare that they have no conflicts of interest with the contents of this article.
