# Supplementary material for: Stable maintenance of MERVL-positive embryonic stem cells reveals sustained transcriptional programs and enhancer remodeling
Source: J Biol Chem. 2026 May 15;302(7):113166. doi: 10.1016/j.jbc.2026.113166 (PMC13273665; doi:10.1016/j.jbc.2026.113166)
Supplement: Supplementary Material [file mmc4.pdf]

SUPPLEMENTARY INFORMATION

Stable maintenance of MERVL-positive embryonic stem cells reveals sustained transcriptional programs and enhancer remodeling

Rui Geng[1-2], Benjamin L. Kidder[1-2*]

[1]Department of Oncology, Wayne State University School of Medicine, Detroit, MI, USA

[2]Karmanos Cancer Institute, Wayne State University School of Medicine, Detroit, MI, USA

Running title: Stable maintenance of MERVL-positive ESCs.

[*]Correspondence:

Benjamin L. Kidder

Email : benjamin.kidder@wayne.edu

Supplemental Figures

Figure S1. Flow cytometry analysis of MERVL expression in ESCs.

Flow cytometry analysis of the 2C::tdTomato reporter to detect MERVL expression in ESCs. Dead cells were excluded using DAPI staining, and live reporter-positive populations are indicated as tdTomato+/DAPI−.

Figure S2. Clonal stability of single-cell-derived Red and Mosaic MERVL-positive ESCs.

(**A**) Flow cytometry analysis of secondary single-cell-derived clones generated from representative Red and Mosaic MERVL-positive ESC lines. Individual tdTomato-positive cells were isolated by FACS, plated at clonal density, expanded, and reanalyzed for MERVL promoter-driven 2C::tdTomato reporter activity. (**B**) Quantification of tdTomato dynamics in secondary clones over time. Curves show total tdTomato-positive area and tdTomato-to-phase area ratios measured by live-cell imaging, with quantitative analysis focused on a 24-hour post-sorting window. (**C**) Representative bright-field and fluorescent images of single-cell-derived Red and Mosaic clones at indicated time points.

Figure S3. Detection and isolation of MERVL reporter-positive cells in R1 ESCs.

(**A**) Flow cytometry analysis of parental R1 ESCs (left) and R1 ESCs following transfection with the MERVL promoter-driven 2C::tdTomato reporter (right). Dead cells were excluded using DAPI staining prior to analysis, and live reporter-positive populations are indicated as tdTomato+/DAPI−. Percentages of tdTomato-positive cells are indicated within gates.

Figure S4. Clonal characterization and live-cell analysis of MERVL reporter-positive R1 ESC lines.

(**A**) Flow cytometry histograms of tdTomato fluorescence intensity in independently derived R1 MERVL reporter-positive clones. Representative Red and Mosaic clones are shown. Percentages indicate the fraction of tdTomato-positive cells within the gated population. (**B**) Representative bright-field and tdTomato fluorescence images of Red and Mosaic R1 reporter-positive clones. (**C**) Quantification of tdTomato signal during live-cell imaging, with analysis focused on a 48-hour interval. Left, tdTomato-positive area normalized to phase area over time for individual clones. Right, cumulative tdTomato signal over time. (**D-E**) Representative live-cell imaging frames of Red (**D**) and Mosaic (**E**) R1 MERVL reporter-positive clones at 0 h and 48 h under self-renewal conditions.

Figure S5. Differential RNA-Seq expression analysis of sMERVL ESCs.

Volcano plot of differentially expressed genes identified between Red and Mosaic sMERVL ESCs and conventional ESCs. Points represent genes, plotted by log2 fold change (log2FC) on the x-axis and p-value significance on the y-axis. Grey points are not significantly differentially expressed (NS), green points signify significant log2FC, blue points indicate significant p-values, and red points mark genes with both significant log2FC and p-values, highlighting the most differentially expressed genes in Red and Mosaic sMERVL ESCs vs. conventional ESC comparison.

Figure S6. MA plots of differential expression in sMERVL ESCs relative to conventional ESCs.

MA-plots were used to compare gene expression in Red and Mosaic sMERVL ESCs to that in conventional ESCs. The plot maps the normalized mean expression levels on the x-axis against the log2 fold changes on the y-axis for each gene. Red dots in the plot denote genes that are significantly downregulated, whereas blue dots represent those that are upregulated.

Figure S7. Clone-level expression of 2C-associated genes in sMERVL ESCs.

(**A**) Dot plot showing mean log2 fold-change (color scale) and statistical significance (dot size, −log10 FDR) for established 2C-associated genes across individual sMERVL ESC clones. Genes are displayed on the y-axis and clones on the x-axis. (**B**) Mean log2 fold-change of selected Zscan4-family genes across individual Mosaic and Red sMERVL ESC clones relative to control ESCs. Bars are grouped by clone and colored by condition.

Figure S8. Gene ontology analysis of differentially expressed genes in sMERVL ESCs.

Clusterprofiler GO term analysis of genes that are upregulated (left; activated) or downregulated (right; suppressed) in Red (left panels) and Mosaic (right panels) sMERVL ESCs compaRed to conventional controls. Enriched GO terms highlight the biological processes and pathways predominantly activated or repressed in these distinct ESC populations, revealing key pathways that are differentially regulated between the cell types.

Figure S9. Gene ontology enrichment and protein interaction networks of genes enriched in Red sMERVL ESCs.

(**A**) Cytoscape visualization of Metascape enrichment analysis, depicting gene ontology clusters specifically activated in Red sMERVL ESCs. (**B**) Protein interaction networks identified by Metascape, illustrating biochemical assemblies and signaling pathways enriched in Red sMERVL ESCs. Nodes are color-coded by gene ontology enrichment p-value, and protein-protein interaction clusters are annotated in corresponding colors.

Figure S10. TRRUST analysis of transcription factor targets in sMERVL ESCs using metascape.

TRRUST analysis identifies and highlights the targets of transcription factors (TFs) whose expression is notably enriched in Red sMERVL ESCs.

Figure S11. Volcano plots and heat map clustering of differentially expressed genes in sMERVL ESCs.

(**A**) Volcano plots display the differential expression of repeat elements (left) and family members (right) between Red and Mosaic sMERVL ESCs compared to conventional ESCs. Each point represents a gene, plotted by log2 fold change (log2FC) on the x-axis and p-value significance on the y-axis. Grey points indicate genes without significant differential expression, green points denote significant log2FC, blue points represent significant p-values, and Red points highlight genes that are significantly different in both log2FC and p-values, showcasing the most distinctively expressed genes in the comparison between Red and Mosaic sMERVL ESCs versus conventional ESCs. Heat maps illustrate

the clustering of differential expression for repeat elements (**B**) and family members (**C**) in Red and Mosaic sMERVL ESCs relative to conventional ESCs, highlighting patterns of gene expression variations across these groups.

Figure S12. Day 12 embryoid body differentiation and morphological segmentation using Detectron2

(**A**) Tile of image segmentation using Detectron2, with each color denoting a different EB. (**B**) Bright field microscopy tiled image displaying the differentiation of EBs at day 12, highlighting Red and Mosaic sMERVL ESCs.

Figure S13. Enrichment of gene ontology (GO) terms in differentiated expressed genes of Red sMERVL ESCs during EB differentiation compaRed to conventional ESCs

Clusterprofiler analysis reveals GO terms enriched in both activated and suppressed genes for groups of 1-5 Red sMERVL ESCs compared to conventional EBs.

Figure S14. Day 12 embryoid body morphology and quantitative segmentation analysis.

(A) Representative bright-field images of day 12 embryoid bodies (EBs) generated from R1 control ESCs and independently derived Red and Mosaic MERVL-positive R1 ESC clones. **(B)** Detectron2-based instance segmentation overlays for corresponding day 12 EBs shown in (A). Individual EBs are segmented and color-coded according to morphology class. **(C)** Quantification of EB morphology distribution at day 12. Top panel,

proportion of EBs by morphology class based on object number. Bottom panel, proportion of total EB area assigned to each morphology class. Morphology categories include solid, cavitated, mixed, and asymmetric.

Figure S15. Day 12 embryoid body differentiation in R1 ESCs and MERVL-positive clones.

(A) Representative bright-field images and corresponding Detectron2 segmentation outputs of day 12 EBs derived from R1 control ESCs and Red and Mosaic MERVL-positive R1 clones. Segmented EBs are shown with color overlays corresponding to classification categories. **(B)** Bright-field images of day 12 EBs derived from R1 control ESCs and independently derived Red and Mosaic MERVL-positive R1 clones.

Figure S16. qRT-PCR analysis of extraembryonic lineage marker expression during EB differentiation.

Quantitative RT-PCR analysis of lineage marker expression in R1 control ESCs and independently derived Red and Mosaic MERVL-positive R1 clones during embryoid body (EB) differentiation. Cells were harvested at day 0 (ESC), day 8 EB, and day 12 EB. Gene expression levels are shown. Expression values are normalized to R1 ESC day 0 levels as indicated on each axis. Bars represent mean ± SEM.

Figure S17. Principal component analysis and H3K27ac differential peak summary.

(A) Principal component analysis (PCA) of bulk RNA-seq samples grouped by condition. Axes represent the first two principal components with the percentage of variance

explained indicated. **(B)** Quantification of H3K27ac differential regions identified by SICER analysis. Left, number of regions with increased H3K27ac signal relative to control ESCs. Right, total number of differential H3K27ac regions identified by SICER.

Figure S18. GREAT analysis of SICER-identified differential H3K4me3 regions in Red and Mosaic MERVL-positive ESCs.

Bubble plot summarizing Gene Ontology (GO) biological process terms identified by GREAT analysis of SICER-defined differential H3K27ac regions between Red MERVL-positive ESCs and control ESCs, and between Mosaic MERVL-positive ESCs and control ESCs. Each column represents an individual sample comparison. Dot size indicates the number of genes assigned to each GO term, and color intensity reflects -log10(q value). Direction of differential enrichment (increased or decreased H3K27ac relative to control ESCs) is indicated below the x-axis.

Figure S19. GREAT analysis of H3K27ac differential regions across individual Red and Mosaic MERVL-positive ESC clones.

Bubble plot showing GO biological process enrichment from GREAT analysis of SICER-identified differential H3K27ac regions for individual Red and Mosaic MERVL-positive ESC clones compared with control ESCs. Each column corresponds to a specific clone-level comparison. Dot size represents the number of genes associated with each GO term, and color intensity corresponds to -log10(q value). Terms are displayed for regions with differential H3K27ac signal relative to control ESCs.

Figure S20. Genome-wide H3K4me3 and H3K27ac density profiles across RefSeq genes.

Average density profiles of (**A**) H3K4me3 and (**B**) H3K27ac across all RefSeq genes, spanning transcription start sites (TSS) to polyadenylation sites (pA). Genes were stratified into quartiles based on expression levels in conventional ESCs, illustrating global patterns of histone modifications and the distinctive epigenetic landscapes of Red and Mosaic sMERVL ESCs.

Figure S21. Epigenome profiling of H3K4me3 and H3K27ac in sMERVL ESCs.

HOMER annotation of (**A**) H3K4me3 and (**B**) H3K27ac peaks in Red and Mosaic sMERVL ESCs, conventional ESCs, and ESCs cultured in differentiation conditions, which revealed enrichment of peaks in promoter, intergenic, and intronic regions. deepTools was used to generate heatmap density profiles of (**C**) H3K4me3 and (**D**) H3K27ac ChIP-Seq signals in ESCs around transcriptional start sites (TSS) and gene body regions generated. Row linked heatmaps show k-means clusters of genes with similar histone modification profiles. Scatter plot of (**E**) H3K4me3 and (**F**) H3K27ac densities in Red and Mosaic sMERVL ESCs relative to conventional ESCs.

Figure S22. Fingerprints of ChIP signal-to-noise ratios.

Deeptools facilitated the generation of ChIP-seq fingerprint profiles, which illustrate the cumulative sum of per-base coverage across each analyzed genomic bin.

Figure S23: Chromatin state analysis and repeat element enrichment in sMERVL ESCs.

(**A**) ChromHMM identification of four chromatin states defined by H3K4me3 and H3K27ac, delineating active regions in sMERVL ESCs. (**B**) ChromHMM enrichment of MERVL-int elements across chromatin states in sMERVL ESCs, conventional ESCs, and ESCs under differentiation conditions. (**C**) ChromHMM enrichment of DNA repeat classes across chromatin states in the same conditions. (**D**) Pearson correlation analysis of ChromHMM enrichment for LTR (top) and LINE (bottom) repeats. (**E**) ChromHMM enrichment of repeat families across chromatin states in ESCs. (**F**) Pearson correlation analysis of ChromHMM enrichment for ERV (top) and ERVL (bottom) repeats.

Figure S24. Pearson correlation of ChromHMM enrichment for DNA repeat across chromatin states in sMERVL ESCs.

Pearson correlation analysis of ChromHMM enrichment analysis of repeat classes in different chromatin states identified in sMERVL ESCs, ESCs, and differentiated cells. The analysis provides insights into the relationships and patterns of chromatin organization associated with repeat elements in these cells.

Figure S25. Pearson correlation of ChromHMM enrichment for DNA family members across chromatin states in ESCs.

Pearson correlation analysis of ChromHMM enrichment analysis of repeat family members in different chromatin states identified in sMERVL ESCs, ESCs, and differentiated cells.

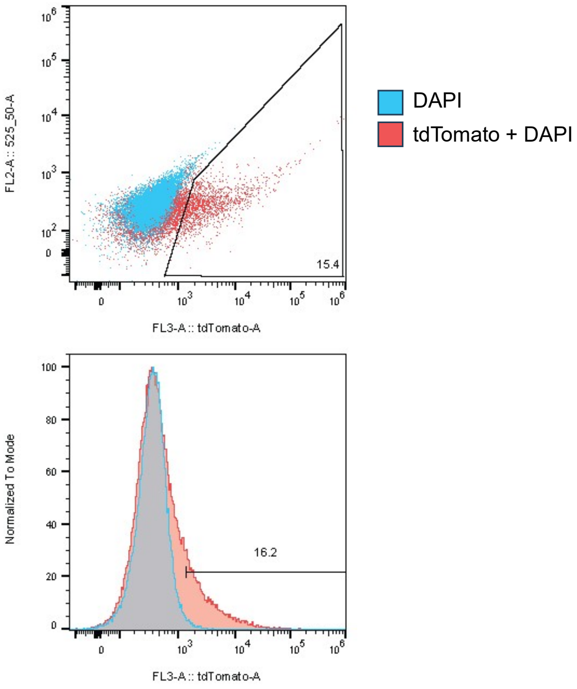
DAPI
tdTomato + DAPI
FL2-A :: 525_50-A
FL3-A :: tdTomato-A
15.4
Normalized To Mode
16.2
FL3-A :: tdTomato-A

Figure S1

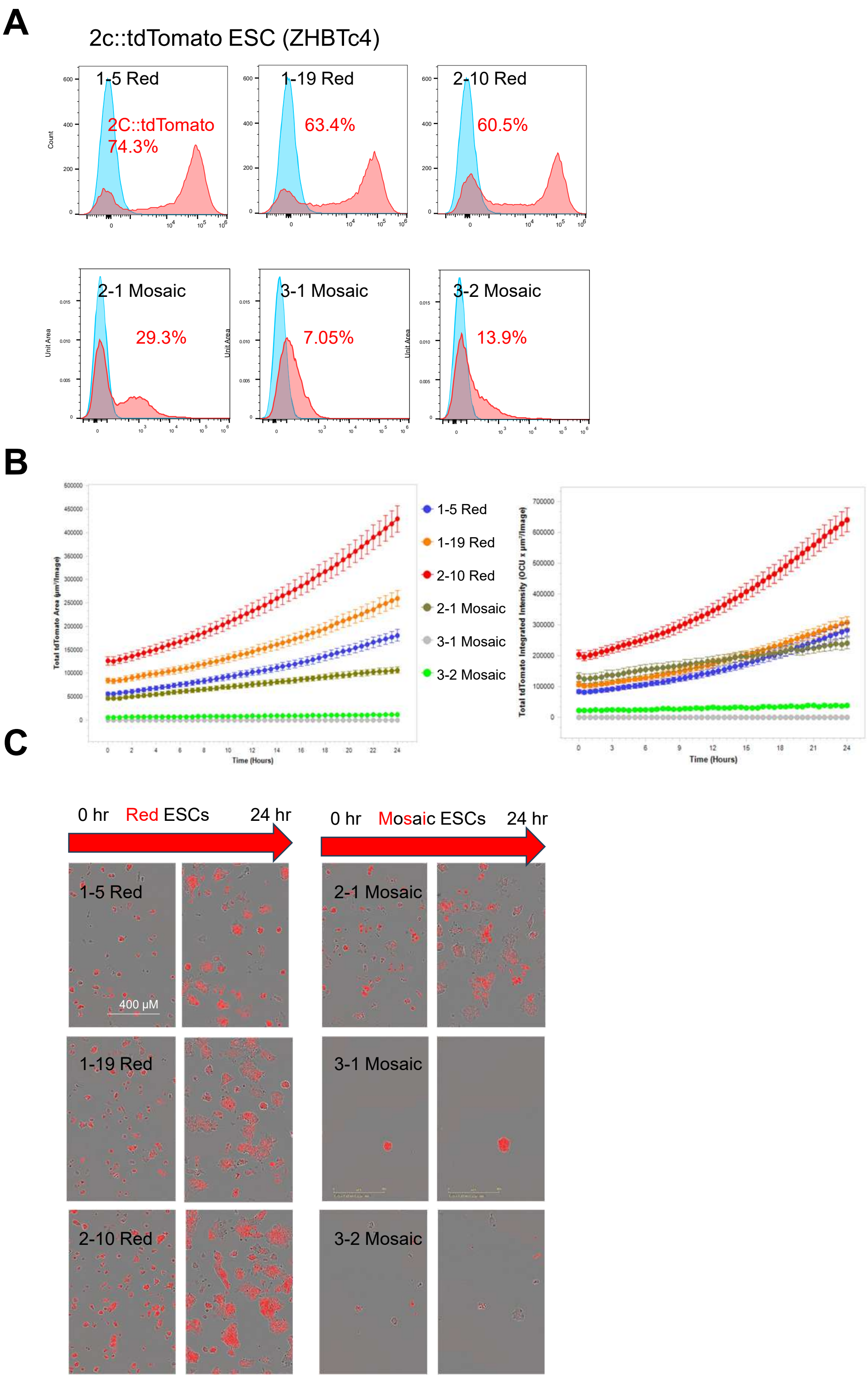
A
2c::tdTomato ESC (ZHBTc4)
1-5 Red
2C::tdTomato
74.3%
1-19 Red
63.4%
2-10 Red
60.5%
2-1 Mosaic
29.3%
3-1 Mosaic
7.05%
3-2 Mosaic
13.9%
B
1-5 Red
1-19 Red
2-10 Red
2-1 Mosaic
3-1 Mosaic
3-2 Mosaic
Time (Hours)
C
0 hr
Red ESCs
24 hr
0 hr
Mosaic ESCs
24 hr
1-5 Red
400 μM
1-19 Red
2-10 Red
2-1 Mosaic
3-1 Mosaic
3-2 Mosaic

Figure S2

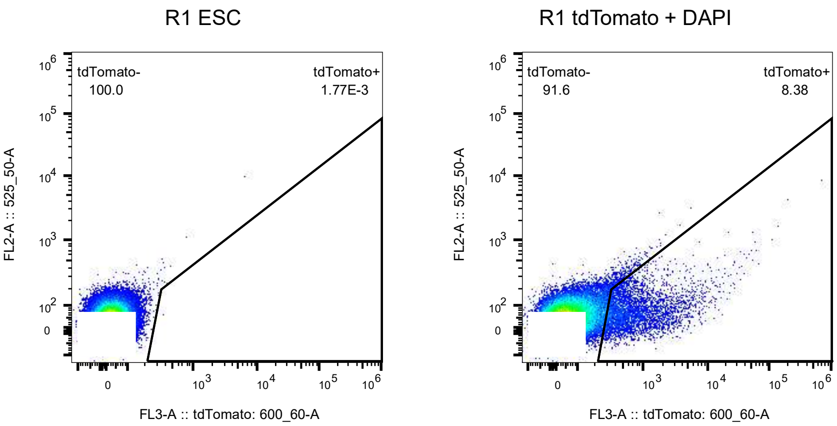
R1 ESC
R1 tdTomato + DAPI
tdTomato-
100.0
tdTomato+
1.77E-3
tdTomato-
91.6
tdTomato+
8.38
FL2-A :: 525_50-A
FL3-A :: tdTomato: 600_60-A

Figure S3

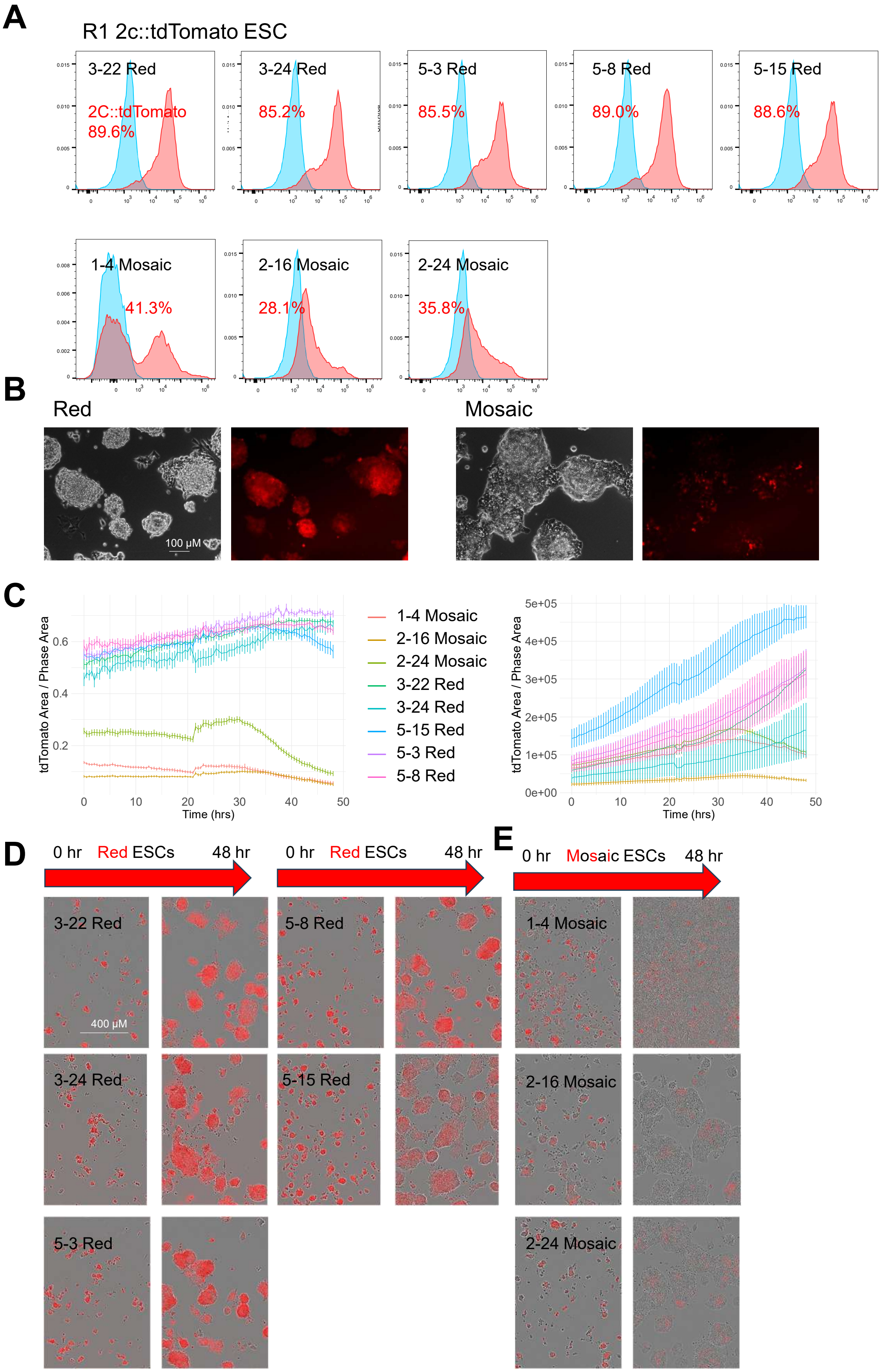
A
R1 2c::tdTomato ESC
3-22 Red
2C::tdTomato 89.6%
3-24 Red
85.2%
5-3 Red
85.5%
5-8 Red
89.0%
5-15 Red
88.6%
1-4 Mosaic
41.3%
2-16 Mosaic
28.1%
2-24 Mosaic
35.8%
B
Red
Mosaic
100 µM
C
tdTomato Area / Phase Area
Time (hrs)
1-4 Mosaic
2-16 Mosaic
2-24 Mosaic
3-22 Red
3-24 Red
5-15 Red
5-3 Red
5-8 Red
D
0 hr Red ESCs 48 hr
0 hr Red ESCs 48 hr
E
0 hr Mosaic ESCs 48 hr
3-22 Red
400 µM
5-8 Red
1-4 Mosaic
3-24 Red
5-15 Red
2-16 Mosaic
5-3 Red
2-24 Mosaic

Figure S4

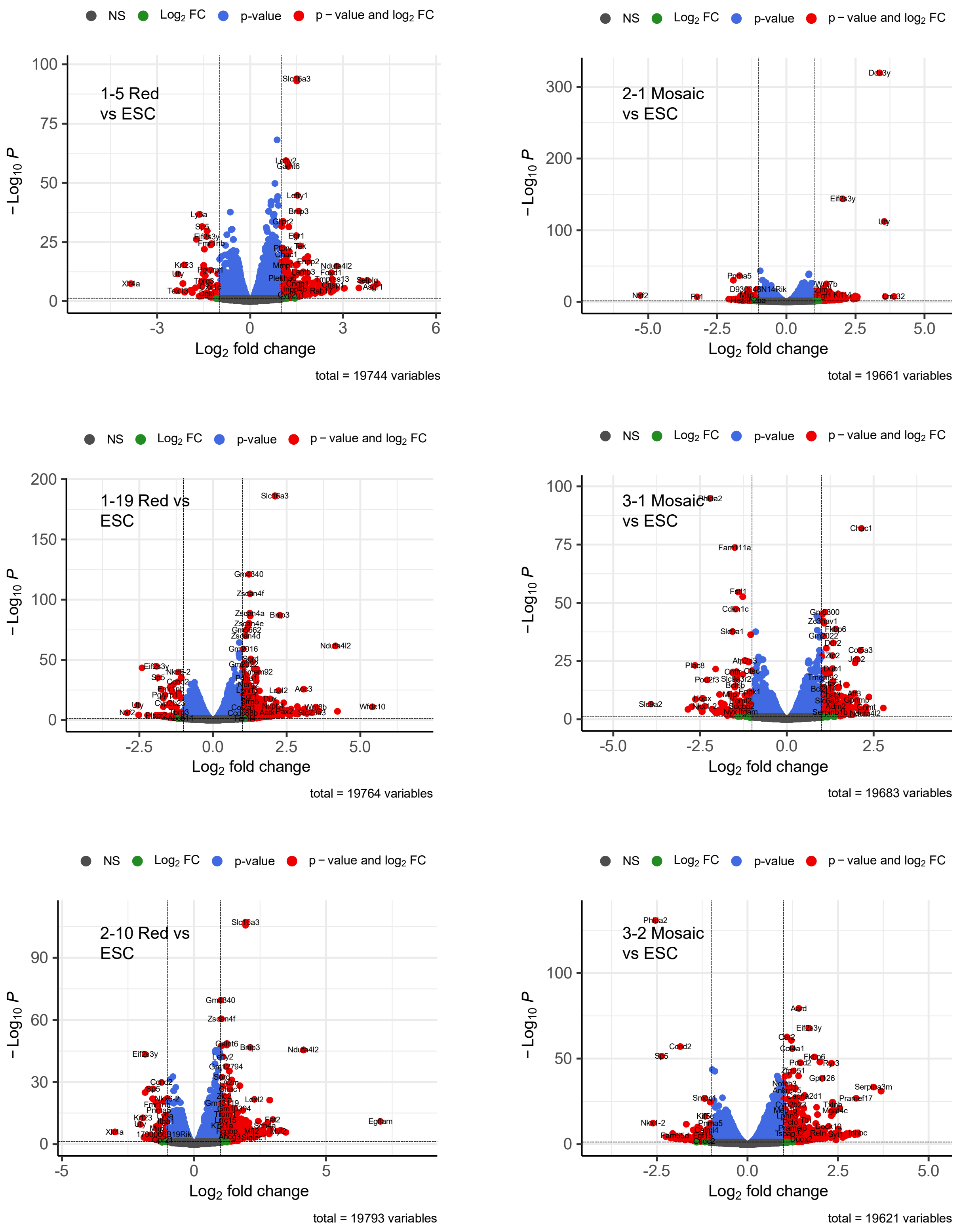
NS
Log2 FC
p-value
p – value and log2 FC
1-5 Red vs ESC
2-1 Mosaic vs ESC
1-19 Red vs ESC
3-1 Mosaic vs ESC
2-10 Red vs ESC
3-2 Mosaic vs ESC
– Log10 P
Log2 fold change
total = 19744 variables
total = 19661 variables
total = 19764 variables
total = 19683 variables
total = 19793 variables
total = 19621 variables

Figure S5

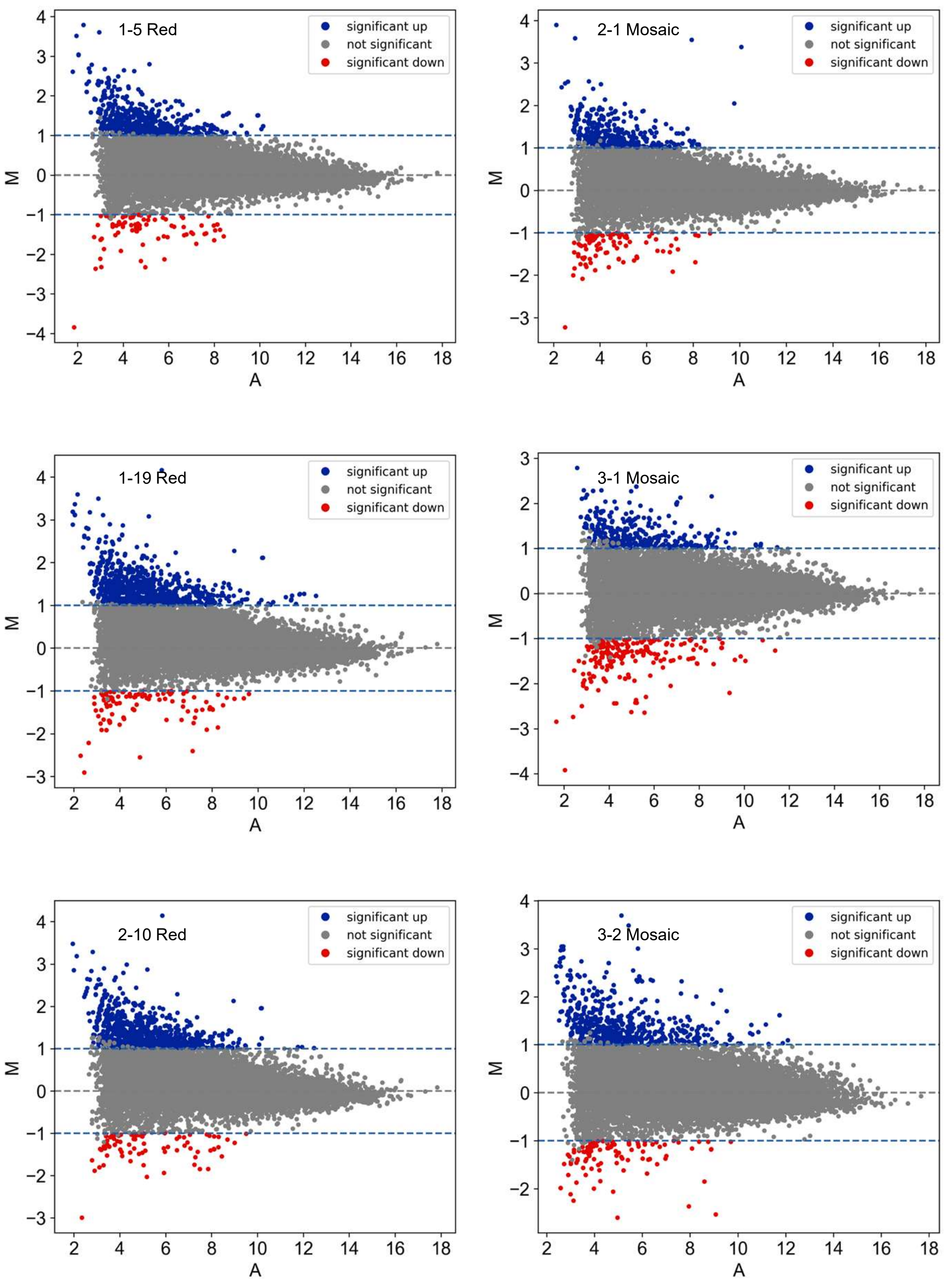

1-5 Red
2-1 Mosaic
1-19 Red
3-1 Mosaic
2-10 Red
3-2 Mosaic
significant up
not significant
significant down
M
A

Figure S6

A Established 2C genes, mean fold-change expression by clone

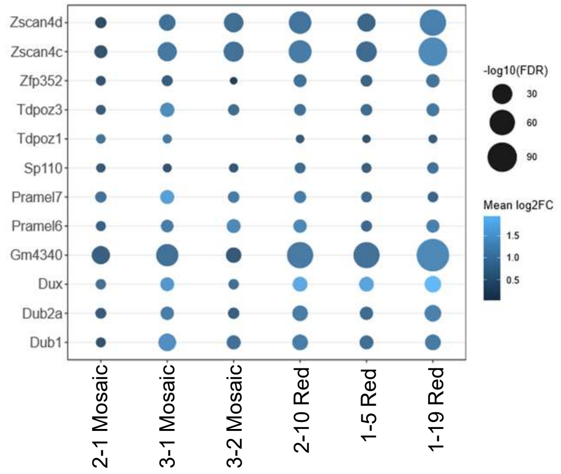

B Zscan4 mean log2 fold-change expression by clone

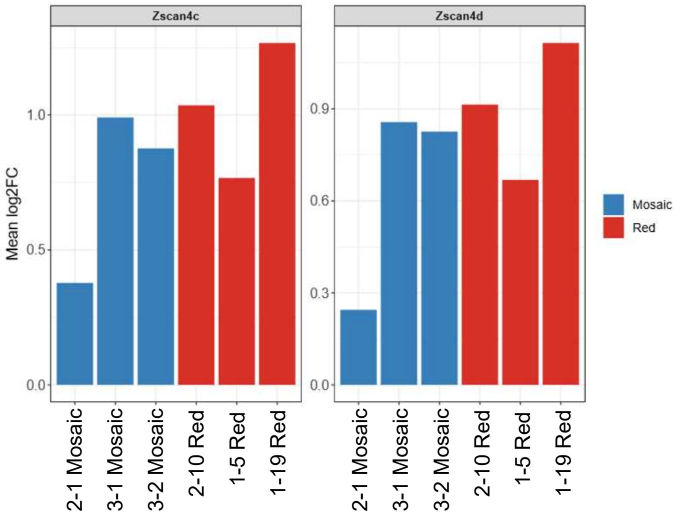

Figure S7

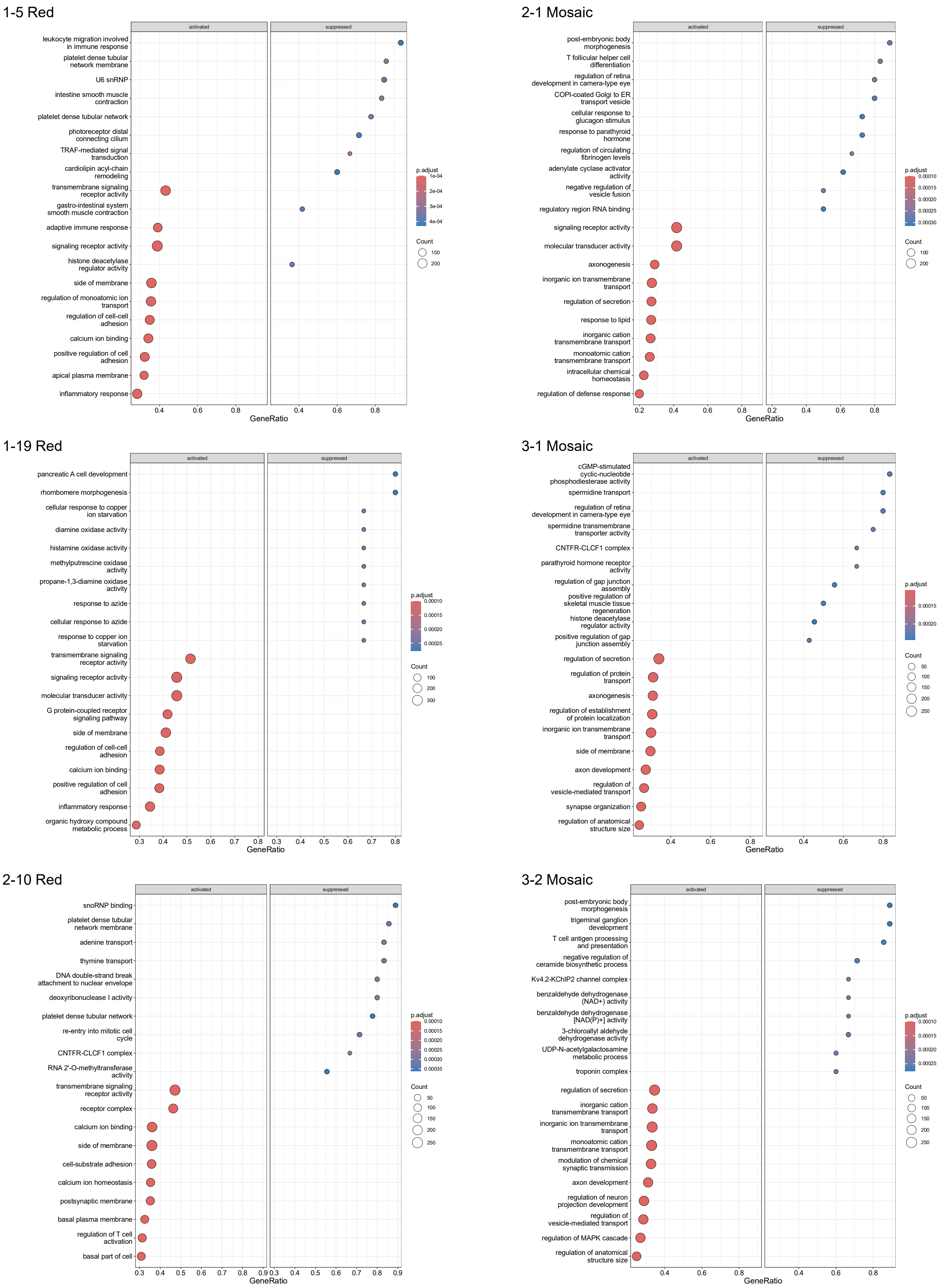
1-5 Red
activated
suppressed
leukocyte migration involved in immune response
platelet dense tubular network membrane
U6 snRNP
intestine smooth muscle contraction
platelet dense tubular network
photoreceptor distal connecting cilium
TRAF-mediated signal transduction
cardiolipin acyl-chain remodeling
transmembrane signaling receptor activity
gastro-intestinal system smooth muscle contraction
adaptive immune response
signaling receptor activity
histone deacetylase regulator activity
side of membrane
regulation of monoatomic ion transport
regulation of cell-cell adhesion
calcium ion binding
positive regulation of cell adhesion
apical plasma membrane
inflammatory response
GeneRatio
p.adjust
Count
2-1 Mosaic
post-embryonic body morphogenesis
T follicular helper cell differentiation
regulation of retina development in camera-type eye
COPI-coated Golgi to ER transport vesicle
cellular response to glucagon stimulus
response to parathyroid hormone
regulation of circulating fibrinogen levels
adenylate cyclase activator activity
negative regulation of vesicle fusion
regulatory region RNA binding
signaling receptor activity
molecular transducer activity
axonogenesis
inorganic ion transmembrane transport
regulation of secretion
response to lipid
inorganic cation transmembrane transport
monoatomic cation transmembrane transport
intracellular chemical homeostasis
regulation of defense response
1-19 Red
pancreatic A cell development
rhombomere morphogenesis
cellular response to copper ion starvation
diamine oxidase activity
histamine oxidase activity
methylputrescine oxidase activity
propane-1,3-diamine oxidase activity
response to azide
cellular response to azide
response to copper ion starvation
transmembrane signaling receptor activity
signaling receptor activity
molecular transducer activity
G protein-coupled receptor signaling pathway
side of membrane
regulation of cell-cell adhesion
calcium ion binding
positive regulation of cell adhesion
inflammatory response
organic hydroxy compound metabolic process
3-1 Mosaic
cGMP-stimulated cyclic-nucleotide phosphodiesterase activity
spermidine transport
regulation of retina development in camera-type eye
spermidine transmembrane transporter activity
CNTFR-CLCF1 complex
parathyroid hormone receptor activity
regulation of gap junction assembly
positive regulation of skeletal muscle tissue regeneration
histone deacetylase regulator activity
positive regulation of gap junction assembly
regulation of secretion
regulation of protein transport
axonogenesis
regulation of establishment of protein localization
inorganic ion transmembrane transport
side of membrane
axon development
regulation of vesicle-mediated transport
synapse organization
regulation of anatomical structure size
2-10 Red
snoRNP binding
platelet dense tubular network membrane
adenine transport
thymine transport
DNA double-strand break attachment to nuclear envelope
deoxyribonuclease I activity
platelet dense tubular network
re-entry into mitotic cell cycle
CNTFR-CLCF1 complex
RNA 2'-O-methyltransferase activity
transmembrane signaling receptor activity
receptor complex
calcium ion binding
side of membrane
cell-substrate adhesion
calcium ion homeostasis
postsynaptic membrane
basal plasma membrane
regulation of T cell activation
basal part of cell
3-2 Mosaic
post-embryonic body morphogenesis
trigeminal ganglion development
T cell antigen processing and presentation
negative regulation of ceramide biosynthetic process
Kv4.2-KChIP2 channel complex
benzaldehyde dehydrogenase (NAD+) activity
benzaldehyde dehydrogenase [NAD(P)+] activity
3-chloroallyl aldehyde dehydrogenase activity
UDP-N-acetylgalactosamine metabolic process
troponin complex
regulation of secretion
inorganic cation transmembrane transport
inorganic ion transmembrane transport
monoatomic cation transmembrane transport
modulation of chemical synaptic transmission
axon development
regulation of neuron projection development
regulation of vesicle-mediated transport
regulation of MAPK cascade
regulation of anatomical structure size

Figure S8

A

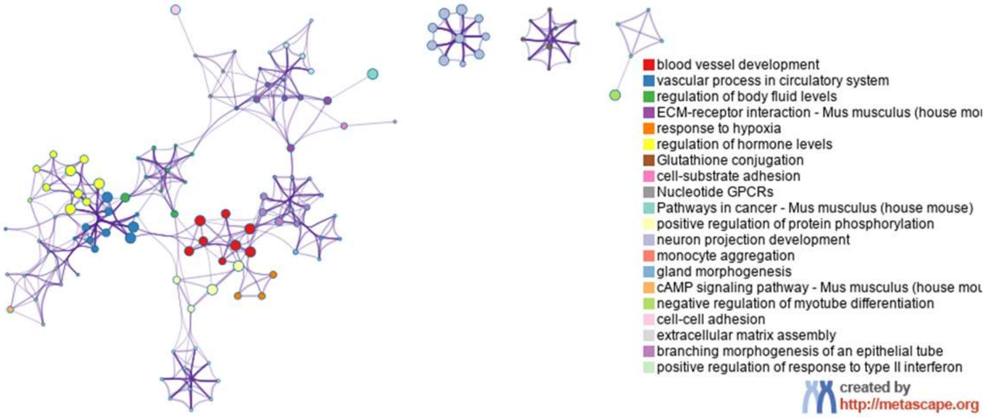
blood vessel development
vascular process in circulatory system
regulation of body fluid levels
ECM-receptor interaction - Mus musculus (house mou
response to hypoxia
regulation of hormone levels
Glutathione conjugation
cell-substrate adhesion
Nucleotide GPCRs
Pathways in cancer - Mus musculus (house mouse)
positive regulation of protein phosphorylation
neuron projection development
monocyte aggregation
gland morphogenesis
cAMP signaling pathway - Mus musculus (house mou
negative regulation of myotube differentiation
cell-cell adhesion
extracellular matrix assembly
branching morphogenesis of an epithelial tube
positive regulation of response to type II interferon
created by
http://metascape.org

B

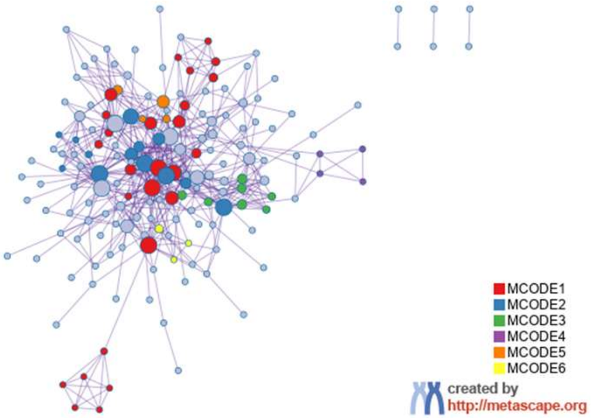
MCODE1
MCODE2
MCODE3
MCODE4
MCODE5
MCODE6
created by
http://metascape.org

Figure S9

Enrichment analysis in TRRUST

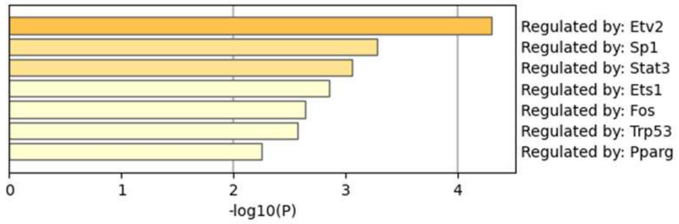

Figure S10

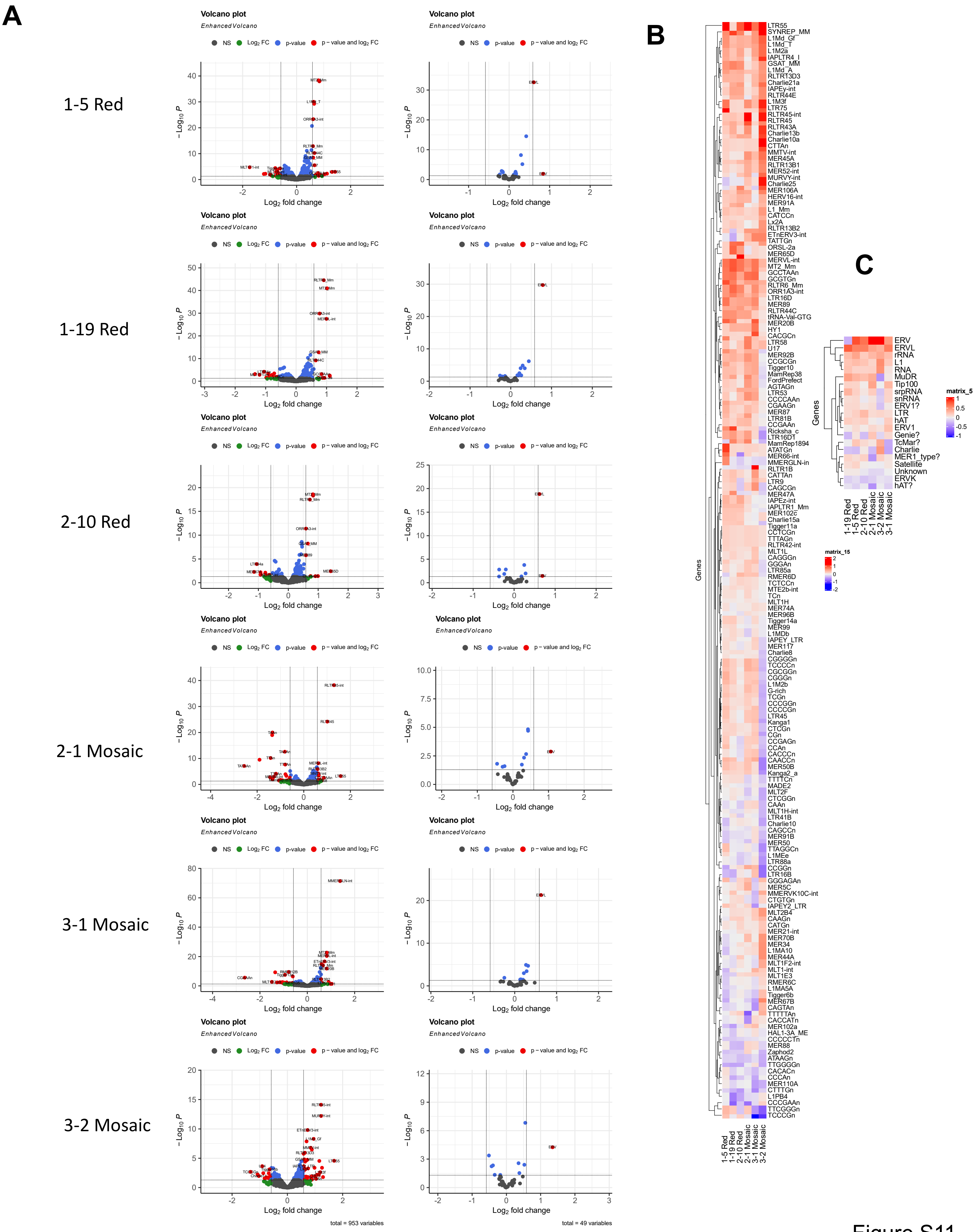
A
B
C
1-5 Red
1-19 Red
2-10 Red
2-1 Mosaic
3-1 Mosaic
3-2 Mosaic
Volcano plot
EnhancedVolcano
NS
Log2 FC
p-value
p – value and log2 FC
Log2 fold change
– Log10 P
total = 953 variables
total = 49 variables
Genes
matrix_15
matrix_5
ERV
ERVL
rRNA
L1
RNA
MuDR
Tip100
srpRNA
snRNA
ERV1?
LTR
hAT
ERV1
Genie?
TcMar?
Charlie
MER1_type?
Satellite
Unknown
ERVK
hAT?
1-19 Red
1-5 Red
2-10 Red
2-1 Mosaic
3-2 Mosaic
3-1 Mosaic

Figure S11

Day 12 EB Differentiation and Segmentation using Detectron2

1-5 Red

1-19 Red

2-10 Red

2-1 Mosaic

3-1 Mosaic

3-2 Mosaic

ESC

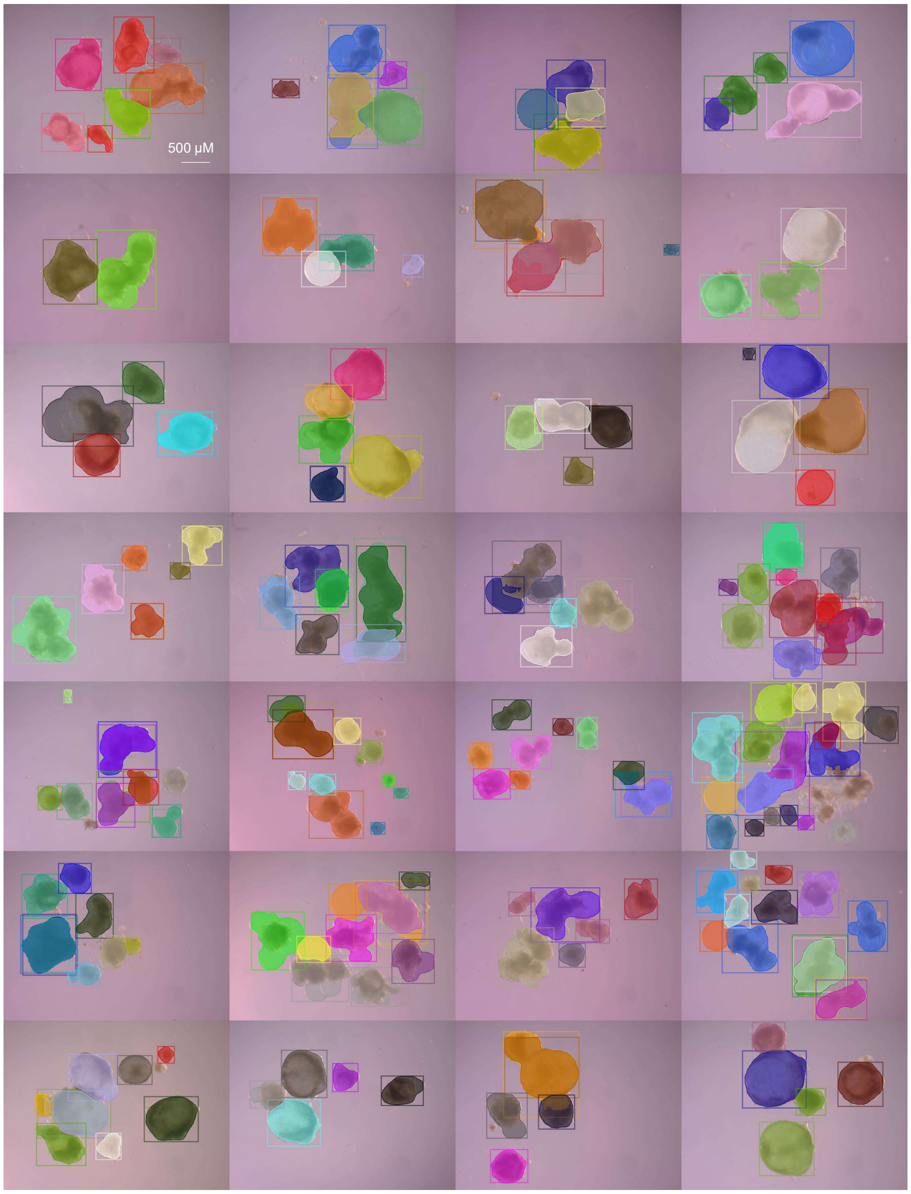

Figure S12A

Day 12 EB Differentiation

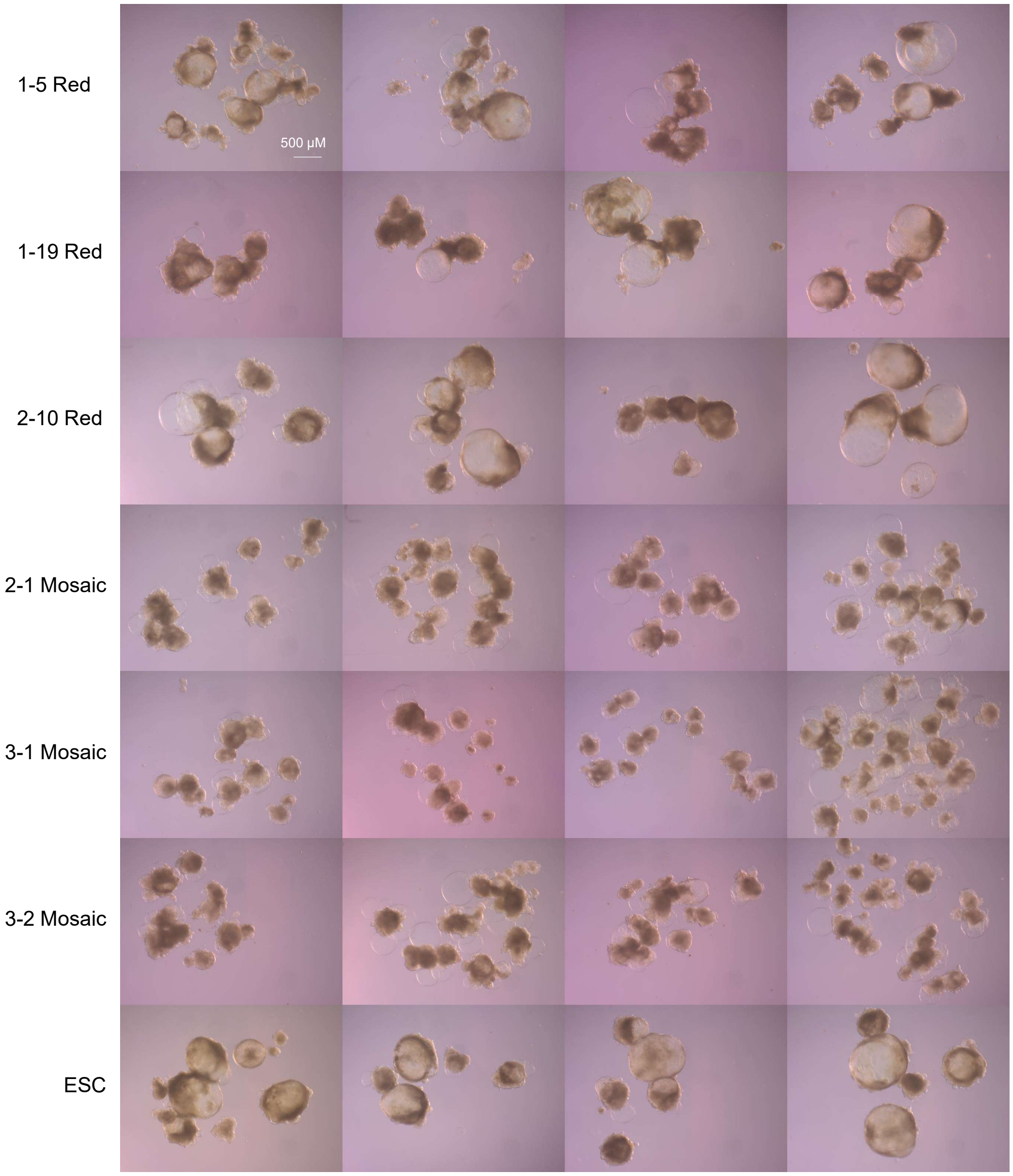

Figure S12B

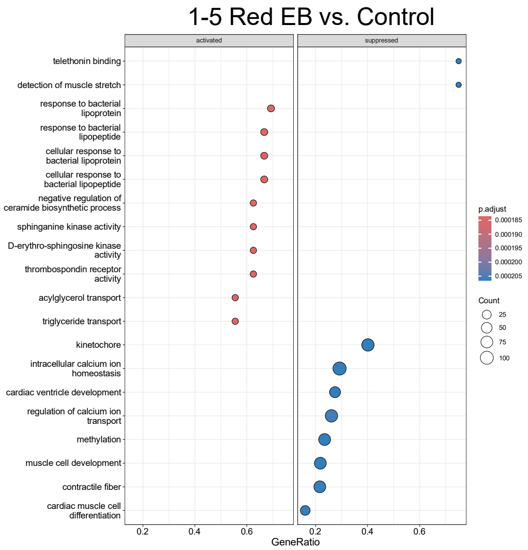

1-5 Red EB vs. Control
activated
suppressed
telethonin binding
detection of muscle stretch
response to bacterial lipoprotein
response to bacterial lipopeptide
cellular response to bacterial lipoprotein
cellular response to bacterial lipopeptide
negative regulation of ceramide biosynthetic process
sphinganine kinase activity
D-erythro-sphingosine kinase activity
thrombospondin receptor activity
acylglycerol transport
triglyceride transport
kinetochore
intracellular calcium ion homeostasis
cardiac ventricle development
regulation of calcium ion transport
methylation
muscle cell development
contractile fiber
cardiac muscle cell differentiation
0.2
0.4
0.6
GeneRatio
p.adjust
0.000185
0.000190
0.000195
0.000200
0.000205
Count
25
50
75
100

Figure S13

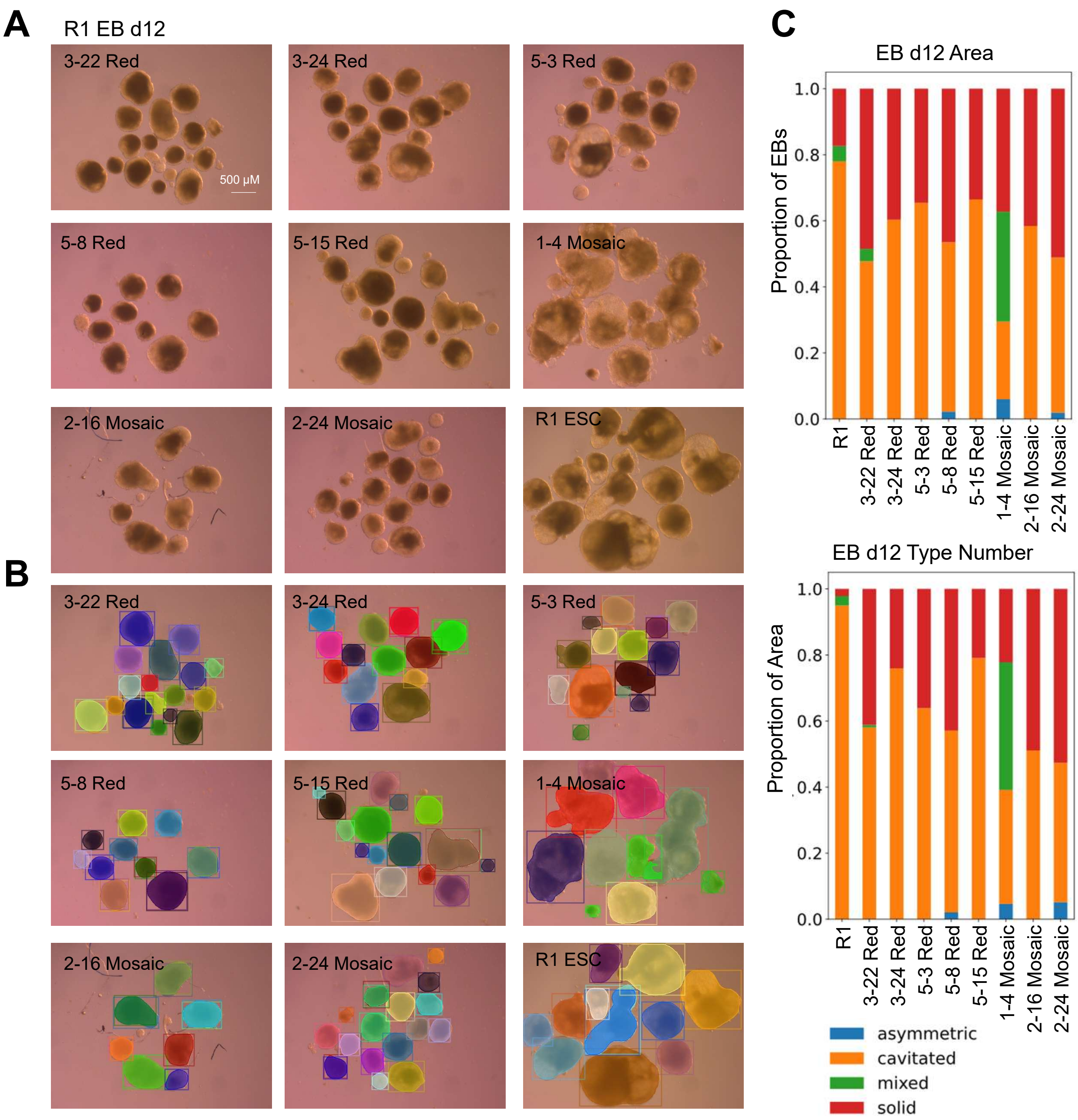
A
R1 EB d12
3-22 Red
3-24 Red
5-3 Red
500 μM
5-8 Red
5-15 Red
1-4 Mosaic
2-16 Mosaic
2-24 Mosaic
R1 ESC
B
C
EB d12 Area
Proportion of EBs
EB d12 Type Number
Proportion of Area
R1
3-22 Red
3-24 Red
5-3 Red
5-8 Red
5-15 Red
1-4 Mosaic
2-16 Mosaic
2-24 Mosaic
asymmetric
cavitated
mixed
solid

Figure S14

Day 12 R1 EB Differentiation and Segmentation using Detectron2

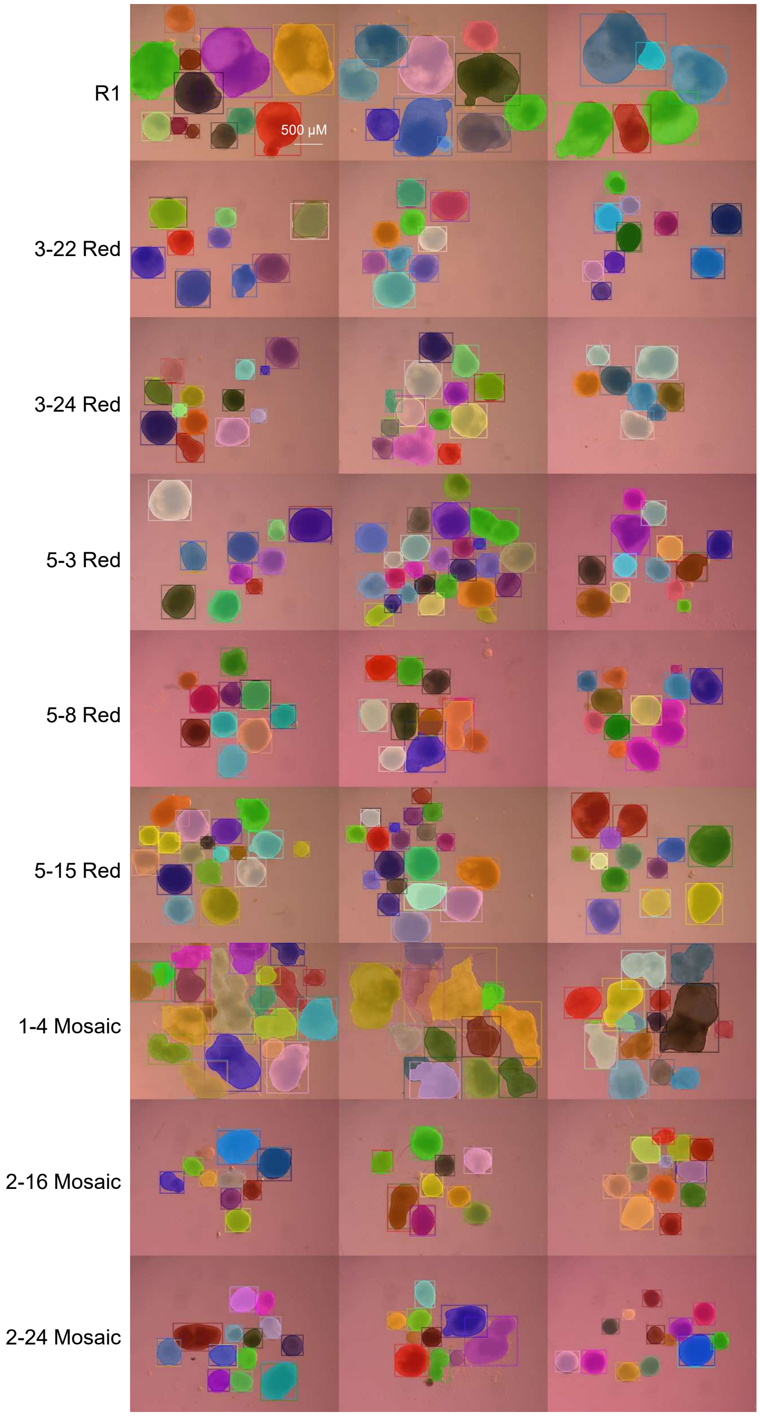

Figure S15A

Day 12 R1 EB Differentiation

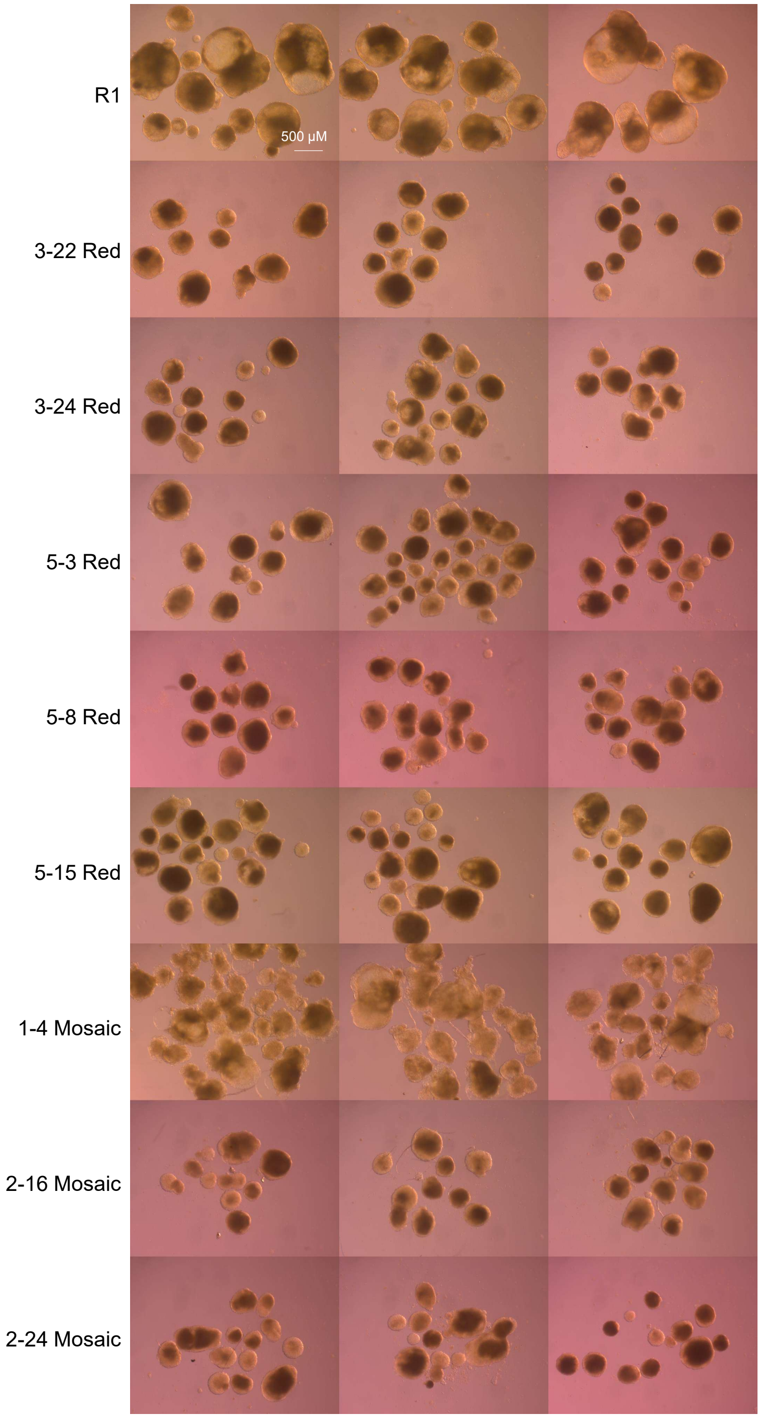

Figure S15B

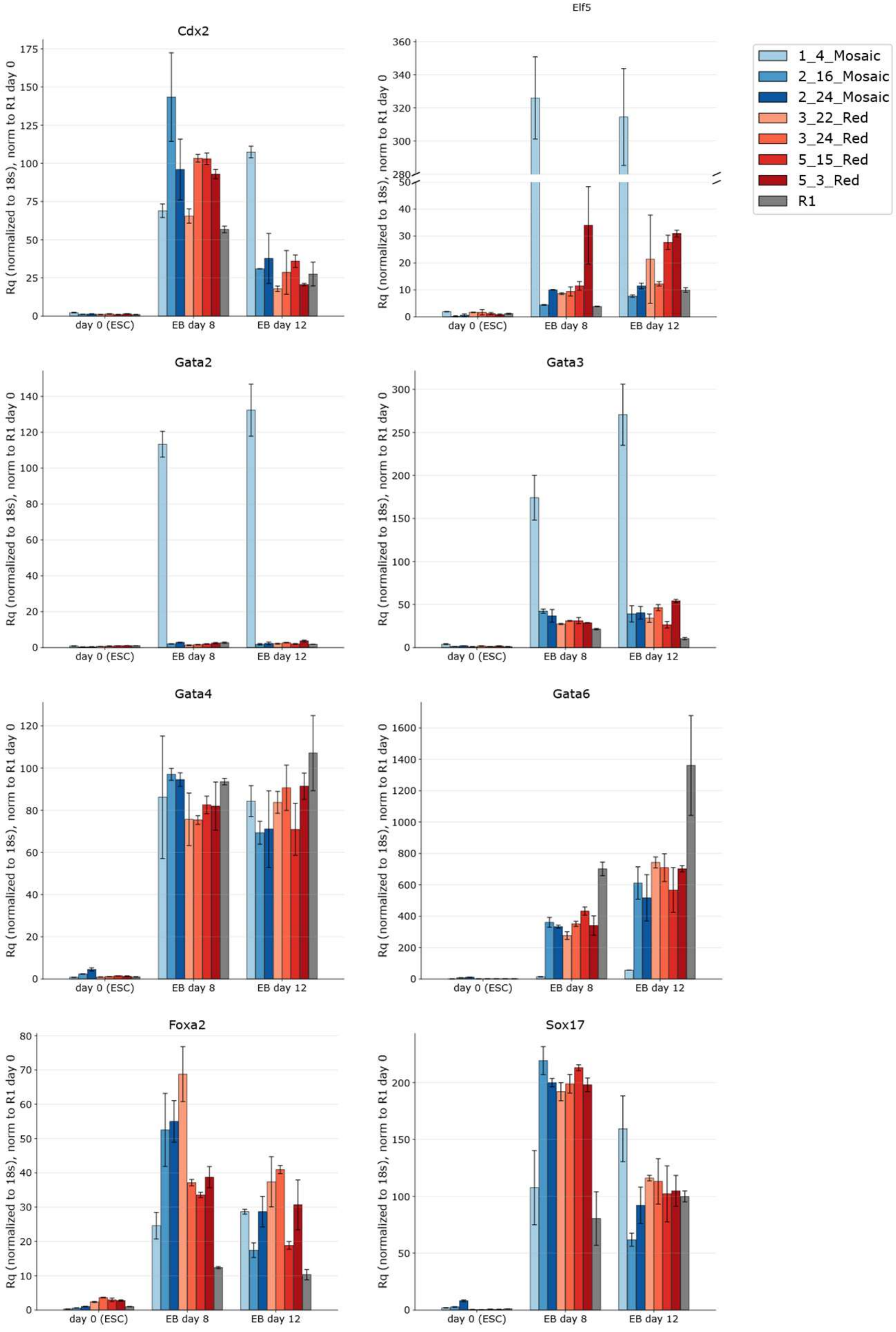
Cdx2
Elf5
Gata2
Gata3
Gata4
Gata6
Foxa2
Sox17
Rq (normalized to 18s), norm to R1 day 0
day 0 (ESC)
EB day 8
EB day 12
1_4_Mosaic
2_16_Mosaic
2_24_Mosaic
3_22_Red
3_24_Red
5_15_Red
5_3_Red
R1

Figure S16

A

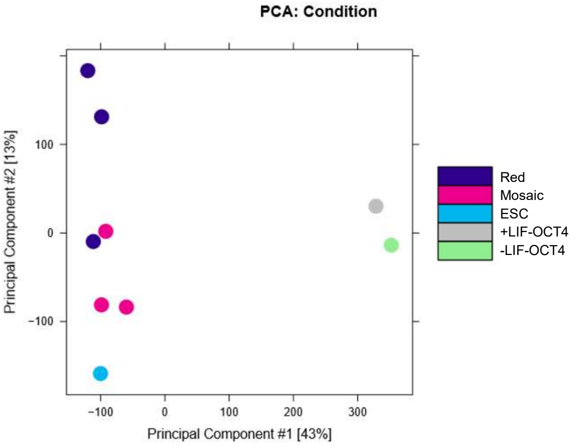
PCA: Condition
Principal Component #2 [13%]
Principal Component #1 [43%]
100
0
−100
−100
0
100
200
300
Red
Mosaic
ESC
+LIF-OCT4
-LIF-OCT4

B

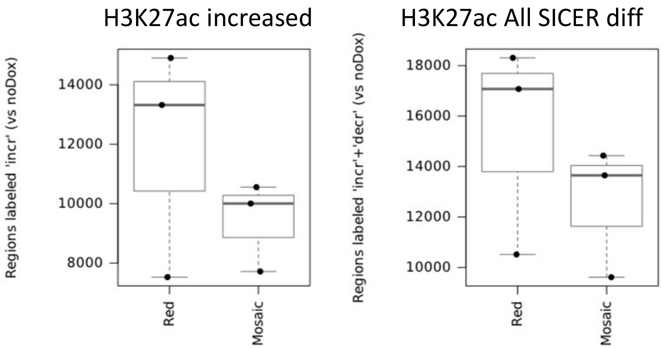
H3K27ac increased
H3K27ac All SICER diff
Regions labeled 'incr' (vs noDox)
Regions labeled 'incr'+'decr' (vs noDox)
14000
12000
10000
8000
18000
16000
14000
12000
10000
Red
Mosaic
Red
Mosaic

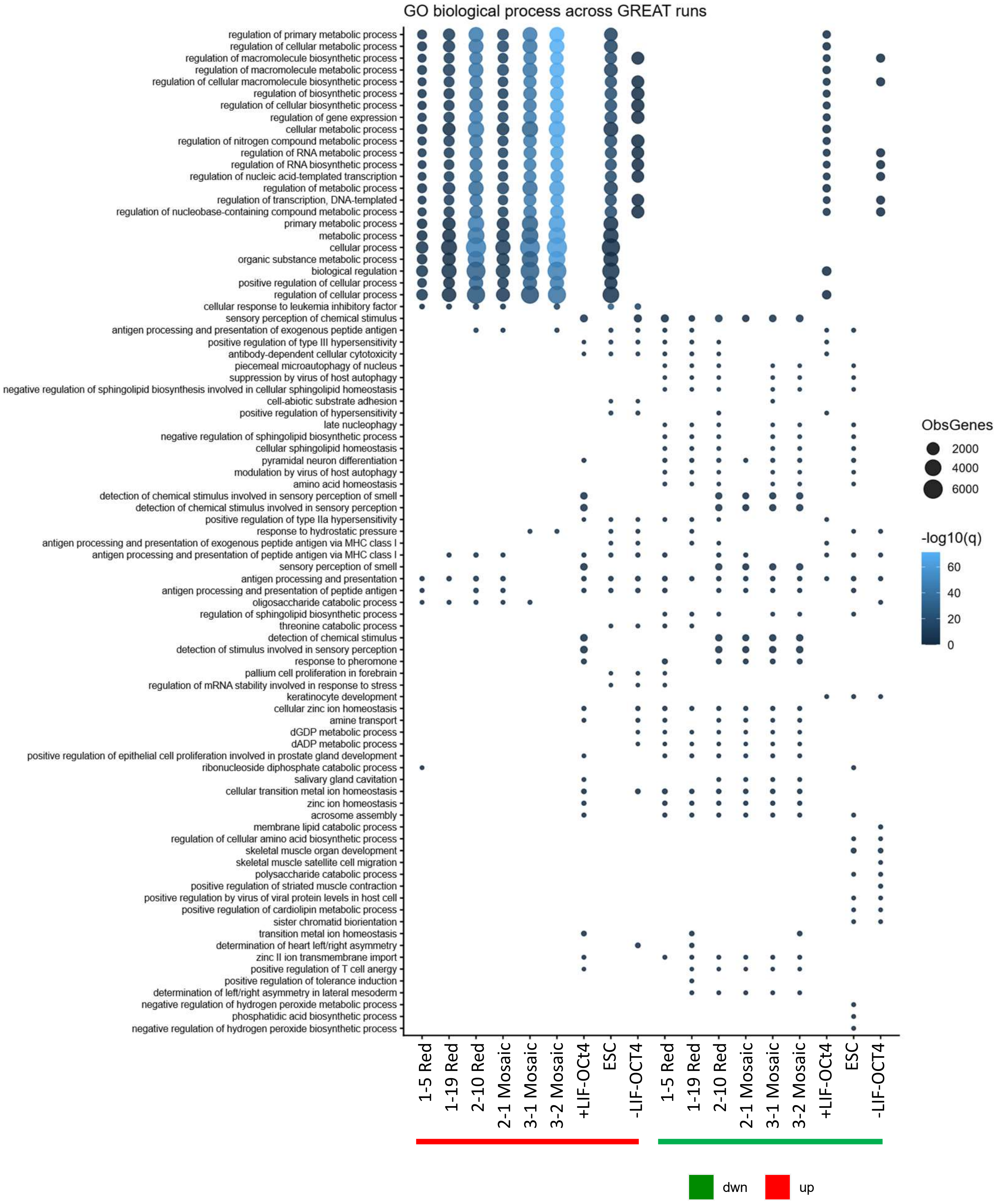
GO biological process across GREAT runs
regulation of primary metabolic process
regulation of cellular metabolic process
regulation of macromolecule biosynthetic process
regulation of macromolecule metabolic process
regulation of cellular macromolecule biosynthetic process
regulation of biosynthetic process
regulation of cellular biosynthetic process
regulation of gene expression
cellular metabolic process
regulation of nitrogen compound metabolic process
regulation of RNA metabolic process
regulation of RNA biosynthetic process
regulation of nucleic acid-templated transcription
regulation of metabolic process
regulation of transcription, DNA-templated
regulation of nucleobase-containing compound metabolic process
primary metabolic process
metabolic process
cellular process
organic substance metabolic process
biological regulation
positive regulation of cellular process
regulation of cellular process
cellular response to leukemia inhibitory factor
sensory perception of chemical stimulus
antigen processing and presentation of exogenous peptide antigen
positive regulation of type III hypersensitivity
antibody-dependent cellular cytotoxicity
piecemeal microautophagy of nucleus
suppression by virus of host autophagy
negative regulation of sphingolipid biosynthesis involved in cellular sphingolipid homeostasis
cell-abiotic substrate adhesion
positive regulation of hypersensitivity
late nucleophagy
negative regulation of sphingolipid biosynthetic process
cellular sphingolipid homeostasis
pyramidal neuron differentiation
modulation by virus of host autophagy
amino acid homeostasis
detection of chemical stimulus involved in sensory perception of smell
detection of chemical stimulus involved in sensory perception
positive regulation of type IIa hypersensitivity
response to hydrostatic pressure
antigen processing and presentation of exogenous peptide antigen via MHC class I
antigen processing and presentation of peptide antigen via MHC class I
sensory perception of smell
antigen processing and presentation
antigen processing and presentation of peptide antigen
oligosaccharide catabolic process
regulation of sphingolipid biosynthetic process
threonine catabolic process
detection of chemical stimulus
detection of stimulus involved in sensory perception
response to pheromone
pallium cell proliferation in forebrain
regulation of mRNA stability involved in response to stress
keratinocyte development
cellular zinc ion homeostasis
amine transport
dGDP metabolic process
dADP metabolic process
positive regulation of epithelial cell proliferation involved in prostate gland development
ribonucleoside diphosphate catabolic process
salivary gland cavitation
cellular transition metal ion homeostasis
zinc ion homeostasis
acrosome assembly
membrane lipid catabolic process
regulation of cellular amino acid biosynthetic process
skeletal muscle organ development
skeletal muscle satellite cell migration
polysaccharide catabolic process
positive regulation of striated muscle contraction
positive regulation by virus of viral protein levels in host cell
positive regulation of cardiolipin metabolic process
sister chromatid biorientation
transition metal ion homeostasis
determination of heart left/right asymmetry
zinc II ion transmembrane import
positive regulation of T cell anergy
positive regulation of tolerance induction
determination of left/right asymmetry in lateral mesoderm
negative regulation of hydrogen peroxide metabolic process
phosphatidic acid biosynthetic process
negative regulation of hydrogen peroxide biosynthetic process
1-5 Red
1-19 Red
2-10 Red
2-1 Mosaic
3-1 Mosaic
3-2 Mosaic
+LIF-OCt4
ESC
-LIF-OCT4
1-5 Red
1-19 Red
2-10 Red
2-1 Mosaic
3-1 Mosaic
3-2 Mosaic
+LIF-OCt4
ESC
-LIF-OCT4
ObsGenes
2000
4000
6000
-log10(q)
60
40
20
0
dwn
up

Figure S18

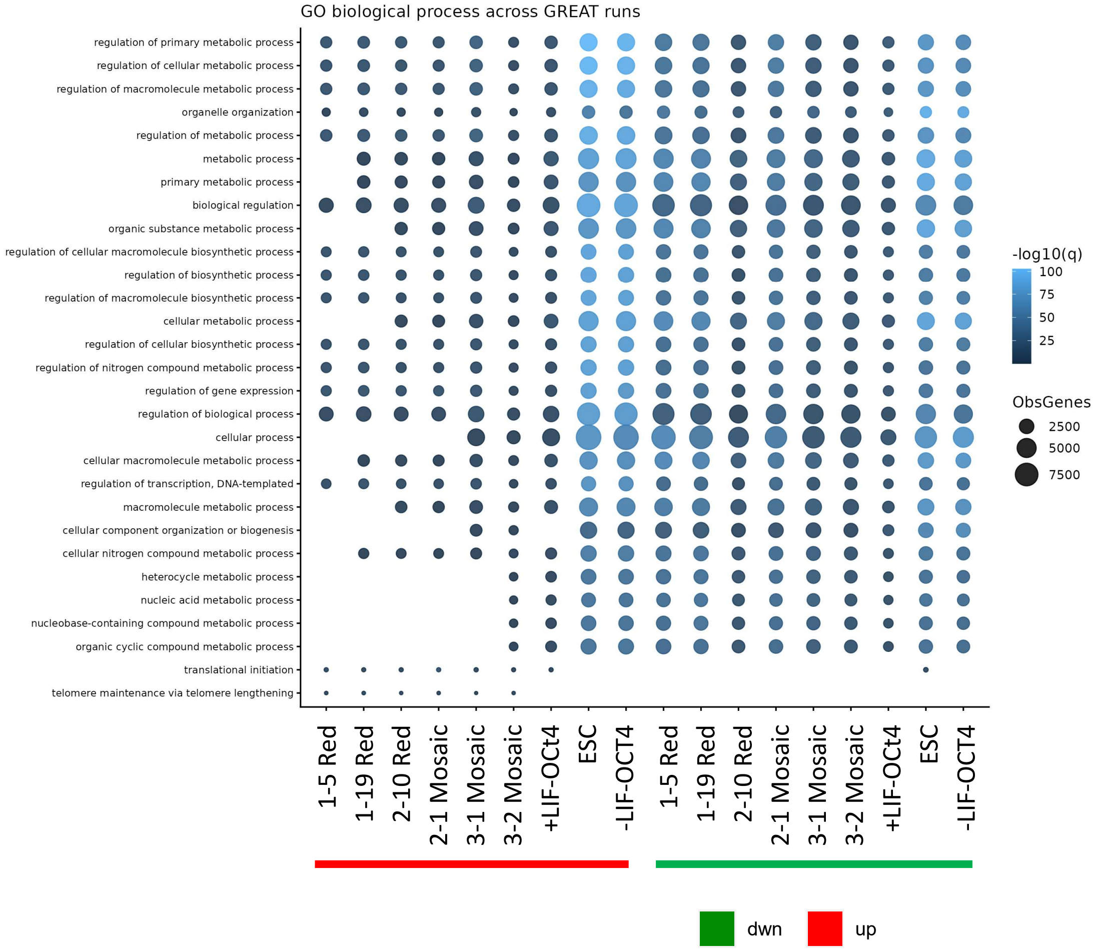
GO biological process across GREAT runs
regulation of primary metabolic process
regulation of cellular metabolic process
regulation of macromolecule metabolic process
organelle organization
regulation of metabolic process
metabolic process
primary metabolic process
biological regulation
organic substance metabolic process
regulation of cellular macromolecule biosynthetic process
regulation of biosynthetic process
regulation of macromolecule biosynthetic process
cellular metabolic process
regulation of cellular biosynthetic process
regulation of nitrogen compound metabolic process
regulation of gene expression
regulation of biological process
cellular process
cellular macromolecule metabolic process
regulation of transcription, DNA-templated
macromolecule metabolic process
cellular component organization or biogenesis
cellular nitrogen compound metabolic process
heterocycle metabolic process
nucleic acid metabolic process
nucleobase-containing compound metabolic process
organic cyclic compound metabolic process
translational initiation
telomere maintenance via telomere lengthening
1-5 Red
1-19 Red
2-10 Red
2-1 Mosaic
3-1 Mosaic
3-2 Mosaic
+LIF-OCt4
ESC
-LIF-OCT4
-log10(q)
100
75
50
25
ObsGenes
2500
5000
7500
dwn
up

Figure S19

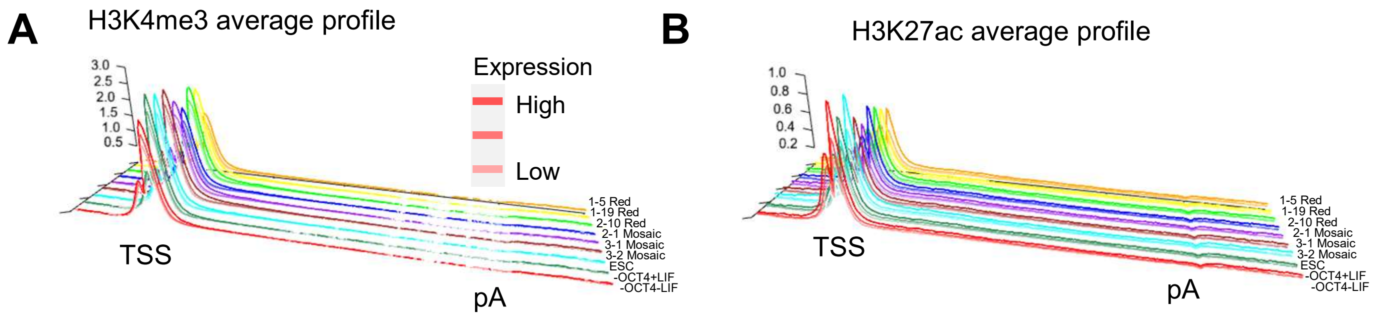
A
H3K4me3 average profile
3.0
2.5
2.0
1.5
1.0
0.5
Expression
High
Low
1-5 Red
1-19 Red
2-10 Red
2-1 Mosaic
3-1 Mosaic
3-2 Mosaic
ESC
-OCT4+LIF
-OCT4-LIF
TSS
pA
B
H3K27ac average profile
1.0
0.8
0.6
0.4
0.2
1-5 Red
1-19 Red
2-10 Red
2-1 Mosaic
3-1 Mosaic
3-2 Mosaic
ESC
-OCT4+LIF
-OCT4-LIF
TSS
pA

Figure S20

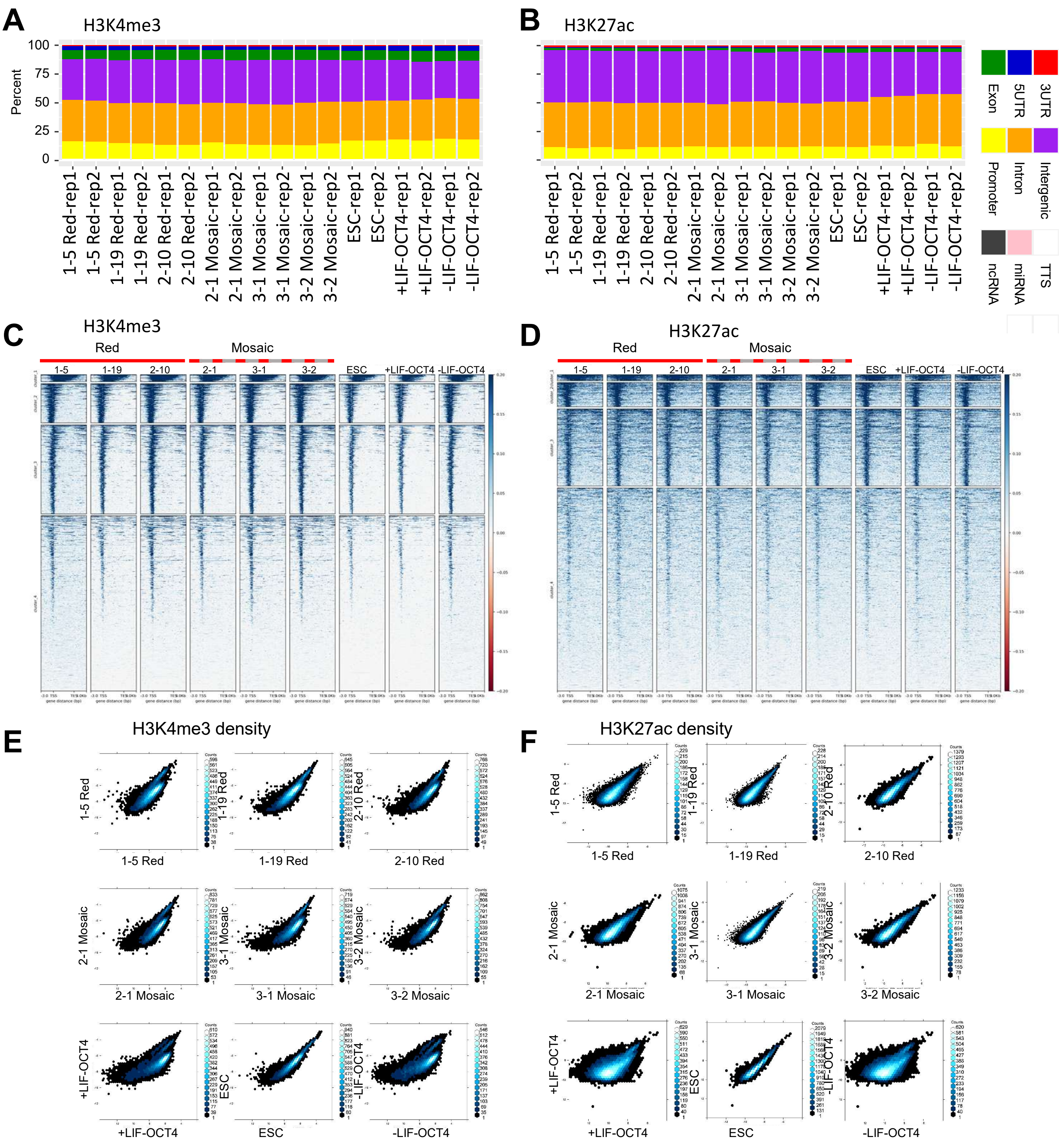
A
H3K4me3
B
H3K27ac
Percent
100
75
50
25
0
1-5 Red-rep1
1-5 Red-rep2
1-19 Red-rep1
1-19 Red-rep2
2-10 Red-rep1
2-10 Red-rep2
2-1 Mosaic-rep1
2-1 Mosaic-rep2
3-1 Mosaic-rep1
3-1 Mosaic-rep2
3-2 Mosaic-rep1
3-2 Mosaic-rep2
ESC-rep1
ESC-rep2
+LIF-OCT4-rep1
+LIF-OCT4-rep2
-LIF-OCT4-rep1
-LIF-OCT4-rep2
Exon
5UTR
3UTR
Promoter
Intron
Intergenic
ncRNA
miRNA
TTS
C
H3K4me3
Red
Mosaic
1-5
1-19
2-10
2-1
3-1
3-2
ESC
+LIF-OCT4
-LIF-OCT4
D
H3K27ac
E
H3K4me3 density
1-5 Red
1-19 Red
2-10 Red
2-1 Mosaic
3-1 Mosaic
3-2 Mosaic
+LIF-OCT4
ESC
-LIF-OCT4
F
H3K27ac density

Figure S21

A

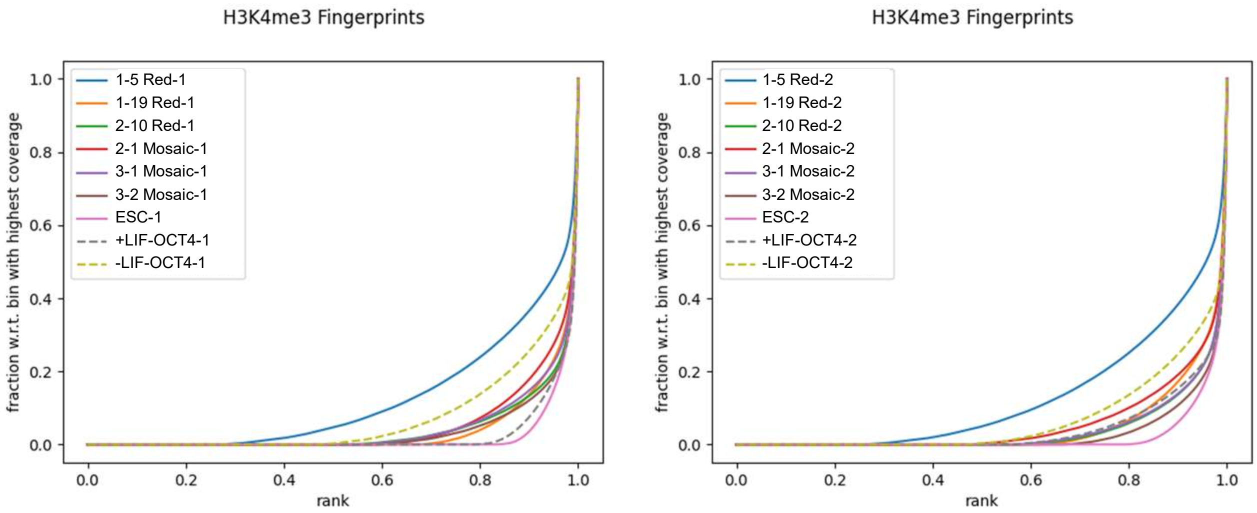

B

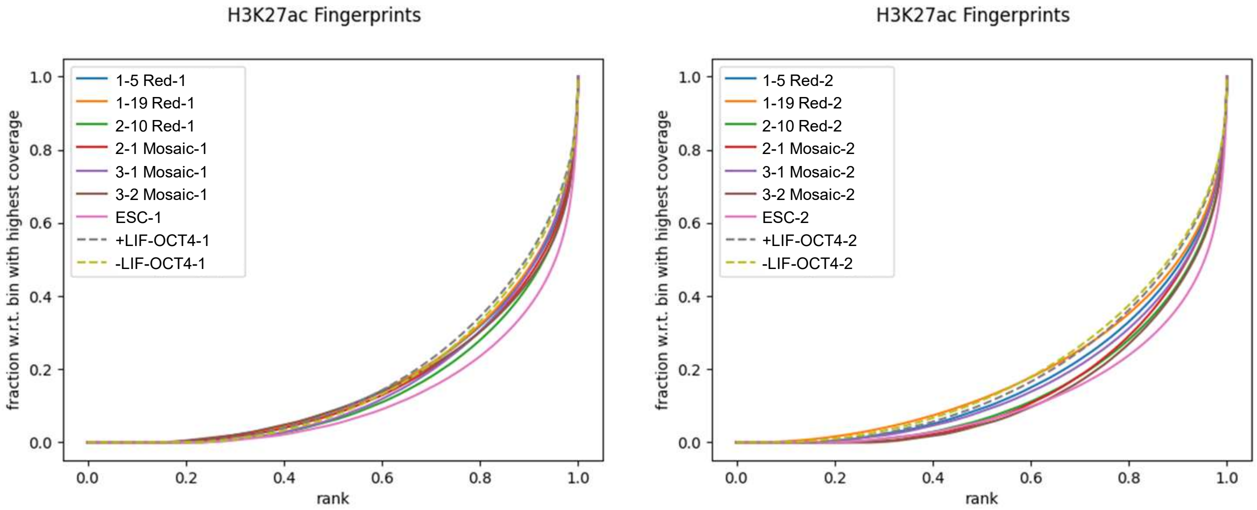

C

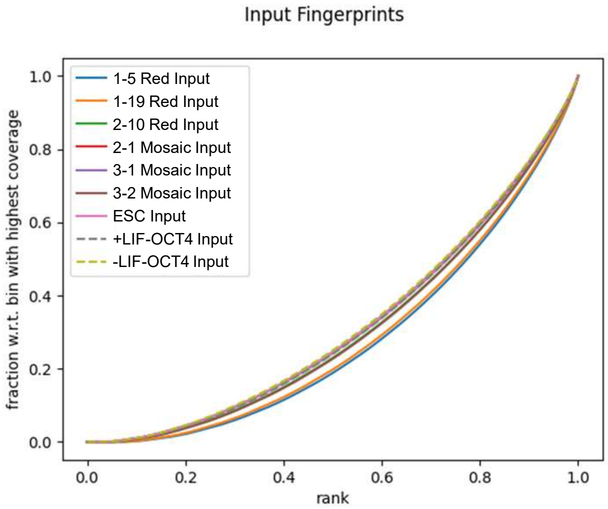

Figure S22

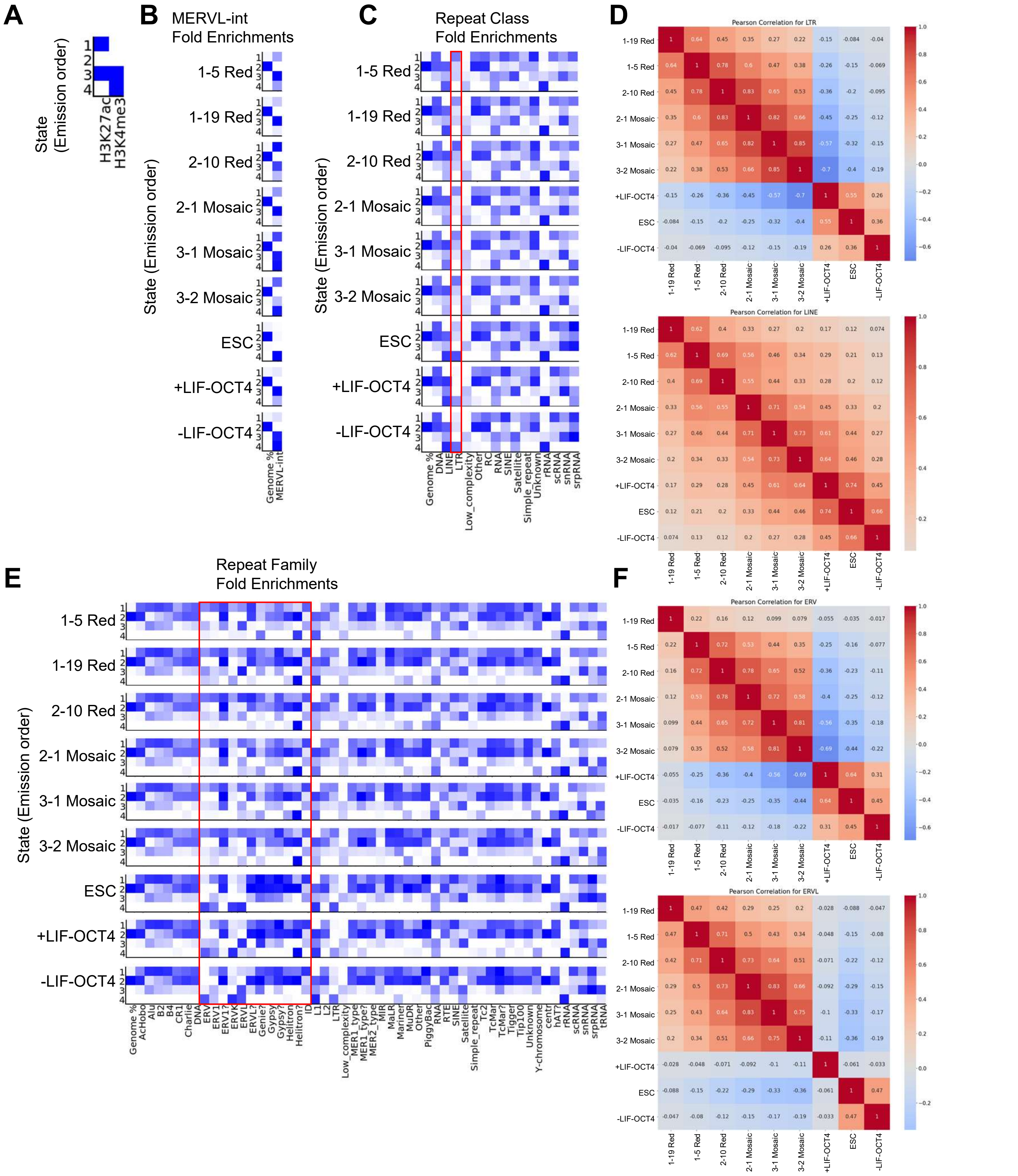
A
State (Emission order)
H3K27ac
H3K4me3
B
MERVL-int Fold Enrichments
1-5 Red
1-19 Red
2-10 Red
2-1 Mosaic
3-1 Mosaic
3-2 Mosaic
ESC
+LIF-OCT4
-LIF-OCT4
Genome %
MERVL-int
C
Repeat Class Fold Enrichments
Genome %
DNA
LINE
LTR
Low_complexity
Other
RC
RNA
SINE
Satellite
Simple_repeat
Unknown
rRNA
scRNA
snRNA
srpRNA
D
Pearson Correlation for LTR
Pearson Correlation for LINE
E
Repeat Family Fold Enrichments
F
Pearson Correlation for ERV
Pearson Correlation for ERVL

Figure S23

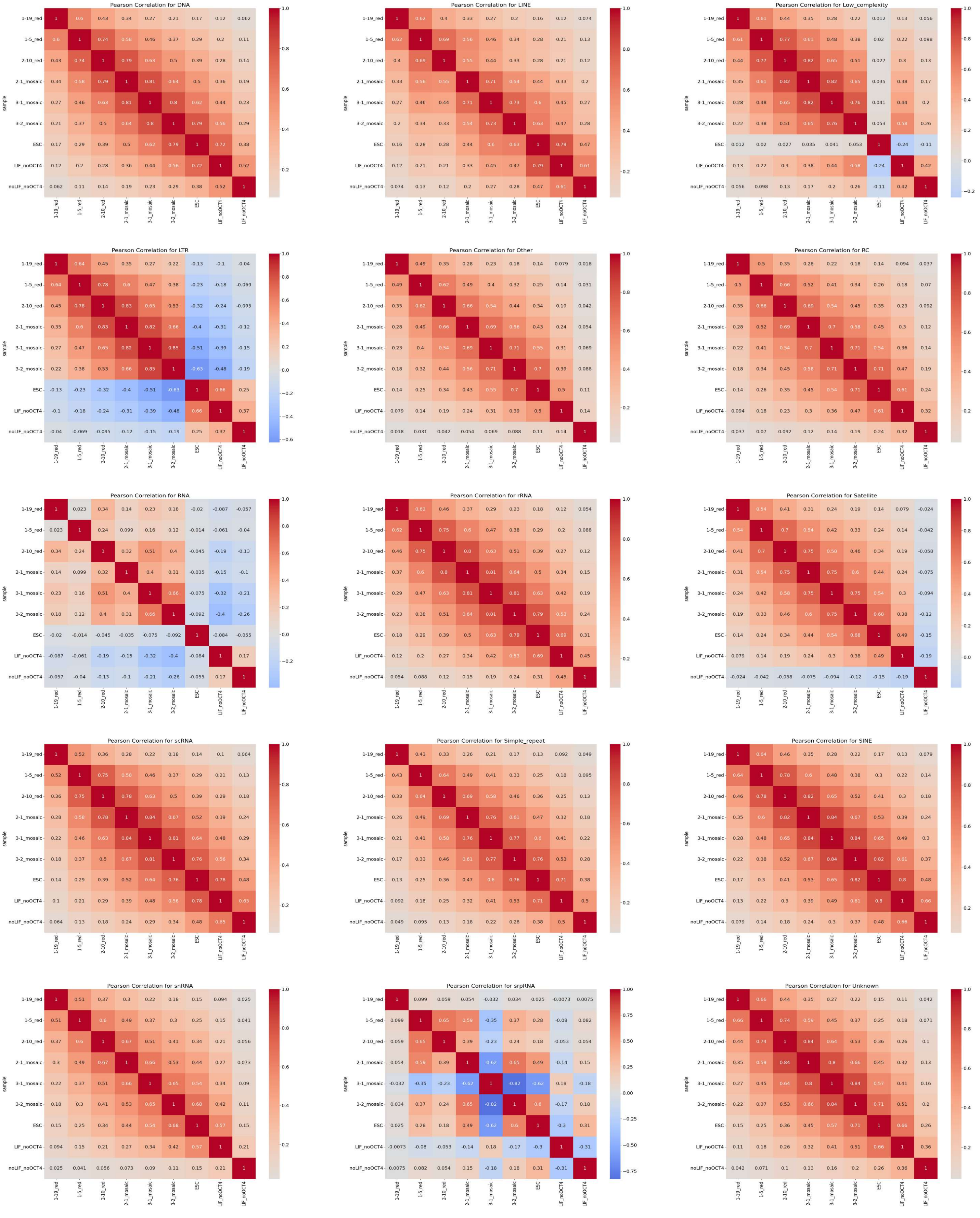
Pearson Correlation for DNA
Pearson Correlation for LINE
Pearson Correlation for Low_complexity
Pearson Correlation for LTR
Pearson Correlation for Other
Pearson Correlation for RC
Pearson Correlation for RNA
Pearson Correlation for rRNA
Pearson Correlation for Satellite
Pearson Correlation for scRNA
Pearson Correlation for Simple_repeat
Pearson Correlation for SINE
Pearson Correlation for snRNA
Pearson Correlation for srpRNA
Pearson Correlation for Unknown
sample
1-19_red
1-5_red
2-10_red
2-1_mosaic
3-1_mosaic
3-2_mosaic
ESC
LIF_noOCT4
noLIF_noOCT4

Figure S24

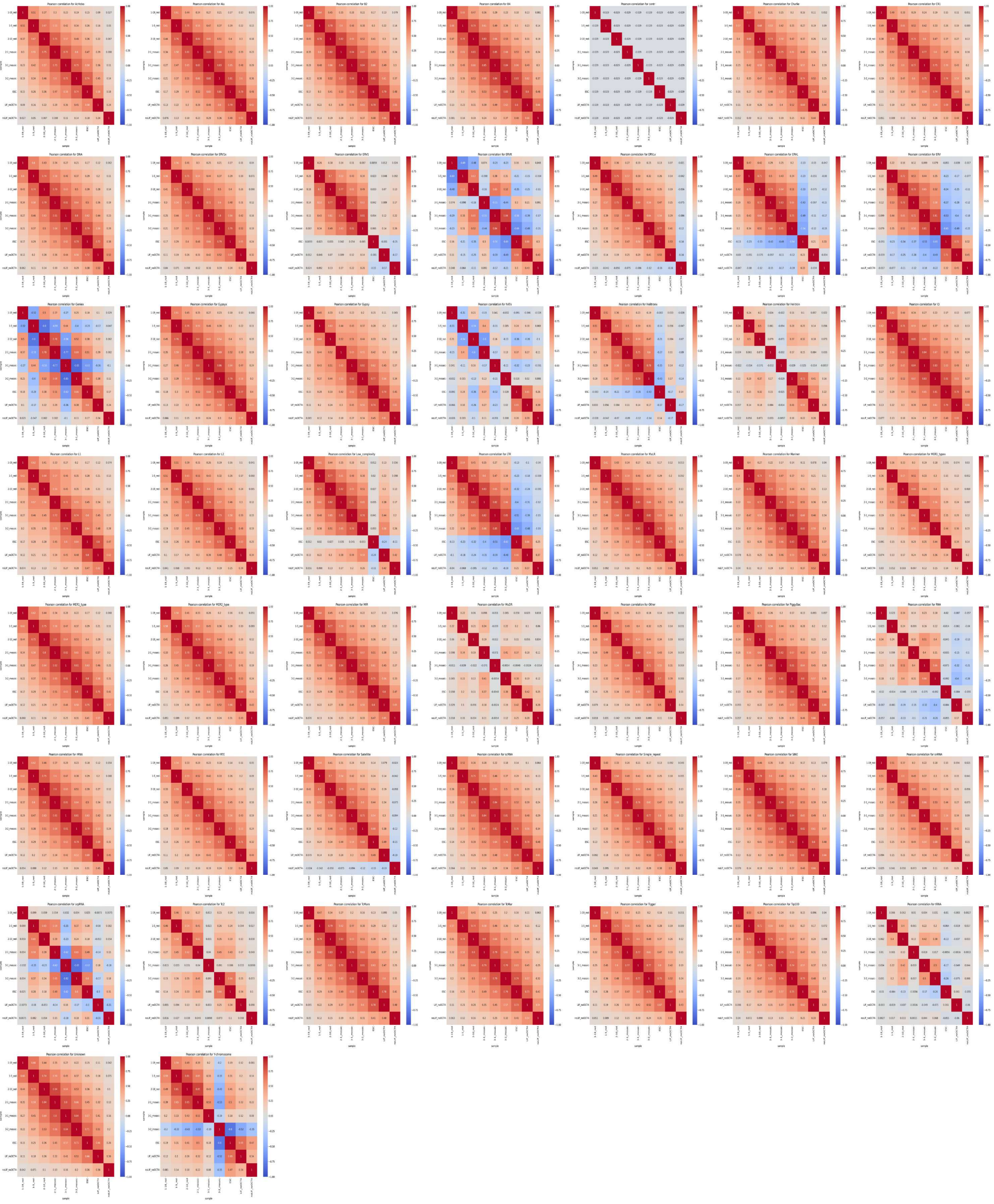

Figure S25
